# A network causal relationship between type-1 diabetes mellitus, 25-hydroxyvitamin D level and systemic lupus erythematosus: Mendelian randomization study

**DOI:** 10.1371/journal.pone.0285915

**Published:** 2023-05-17

**Authors:** Kaisheng Su, Zhifang Jia, Yanhua Wu, Yuanlin Sun, Qi Gao, Zhenyu Jiang, Jing Jiang

**Affiliations:** 1 Department of Epidemiology and Biostatistics, School of Public Health, Jilin University, Changchun, Jilin Province, China; 2 Department of Clinical Epidemiology, The First Hospital of Jilin University, Changchun, Jilin Province, China; 3 Department of Gastrointestinal Surgery, The First Hospital of Jilin University, Changchun, China; 4 Department of Rheumatology, The First Hospital of Jilin University, Changchun, Jilin Province, China; Shenzhen Baoan Women’s and Children’s Hospital, CHINA

## Abstract

**Background:**

Observational studies have suggested a relationship between type-1 diabetes mellitus (T1DM) and systemic lupus erythematosus (SLE). In both autoimmunities, 25-hydroxyvitamin D (25-OHD) deficiency is common. However, the causality between T1DM, 25-OHD level and SLE remains largely unknown.

**Methods:**

Independent genetic variants associated with T1DM, 25-OHD level, and SLE from the largest genome-wide association studies were used to conduct two-sample bidirectional Mendelian randomization (BIMR) and two-step Mendelian randomization (MR) analysis to estimate causal relationship between T1DM, 25-OHD level and SLE, and further multivariable Mendelian randomization (MVMR) was used to verify direct causality of T1DM and 25-OHD level on SLE. A series of sensitivity analysis as validation of primary MR results were performed.

**Results:**

Consistent with the results of BIMR, there was strong evidence for a direct causal effect of T1DM on the risk of SLE (OR_MVMR-IVW_ = 1.249, 95% CI = 1.148–1.360, *P*_MVMR-IVW_ = 1.25×10^−5^), and 25-OHD level was negatively associated with the risk of SLE (OR_MVMR-IVW_ = 0.305, 95% CI = 0.109–0.857, *P*_MVMR-IVW_ = 0.031). We also observed a negative causal effect of T1DM on 25-OHD level (OR_BIMR-IVW_ = 0.995, 95% CI = 0.991–0.999, *P*_BIMR-IVW_ = 0.030) while the causal effect of 25-OHD level on the risk of T1DM did not exist (*P*_BIMR-IVW_ = 0.106). In BIMR analysis, there was no evidence for causal effects of SLE on the risk of T1DM and 25-OHD level (*P*_BIMR-IVW_ > 0.05, respectively).

**Conclusion:**

Our MR analysis suggested that there was a network causal relationship between T1DM, 25-OHD level and SLE. T1DM and 25-OHD level both have causal associations with the risk of SLE, and 25-OHD level could be a mediator in the causality of T1DM and SLE.

## Introduction

Autoimmune diseases can be classified into two types according to the range of autoimmunity: organ-specific autoimmunity involves only specific organs (cells), such as type I diabetes mellitus (T1DM), and systematic autoimmunity involves multiple systems, such as systematic lupus erythematosus (SLE). The coexistence of organ-specific autoimmunity and systematic autoimmunity within the same patient is a common clinical phenomenon, for instance, Hashimoto’s thyroiditis (HT) /T1DM and SLE [1, 2]. However, the pathogenesis and directional relationship of such coexistence remain unclear. Systematic lupus erythematosus (SLE) is a chronic systematic autoimmune disease characterized by multi-organ functional impairment resulting from the disorder of immunoregulation and immune tolerance of both the natural and acquired immune systems [3]. T1DM is a chronic disease characterized by insulin deficiency due to autoimmune pancreatic β-cell loss and leads to hyperglycaemia, in which the significant pathogenesis of B-cell-mediated autoantibody production and T-cell-mediated autoimmune cytotoxic effects was similar to SLE [4]. Detected β-cell targeting autoantibodies, presymptomatic and symptomatic T1DM in young SLE patients are not uncommon [2]. The two different types of autoimmunity not only have clinical associations but also similar pathogeneses: autoantibodies, activated immune cells and the activation of inflammatory pathways [3, 4]. Autoimmune diseases are constantly changing due to the combined effects of susceptibility genes and environmental triggers, and multiple autoimmunity is also prone to occur [5]. With the intriguing question of the mechanism of such disease conversion and coexistence, clarifying the directional relationship among multiple autoimmunity is the first link to be answered.

Environmental triggers plays a crucial role in the etiology of autoimmunity, immunoregulatory dysfunction caused by 25-hydroxyvitamin D (25-OHD) deficiency is considered a possible environmental trigger for multiple autoimmune diseases [5–7]. 25-OHD can regulate the adaptive immune system through the vitamin D receptor (VDR) expressed on the surface of activated antigen-presenting cell (APC), T cells and B cells, which could lead to the transition of the immune system from immune proinflammatory to immune tolerance [8]. Several observational studies have confirmed that 25-OHD deficiency was common in both T1DM and SLE, while the causality remains unclear [9, 10]. As a possible environmental trigger for autoimmunity [5, 11], it is important to know the causality between 25-OHD deficiency and organ-specific autoimmunity/systematic autoimmunity, as well as the role of 25-OHD in the multiple autoimmunity, in order to further clarify the pathogenesis, disease prevention, and prognosis improvement.

**Fig 1:**
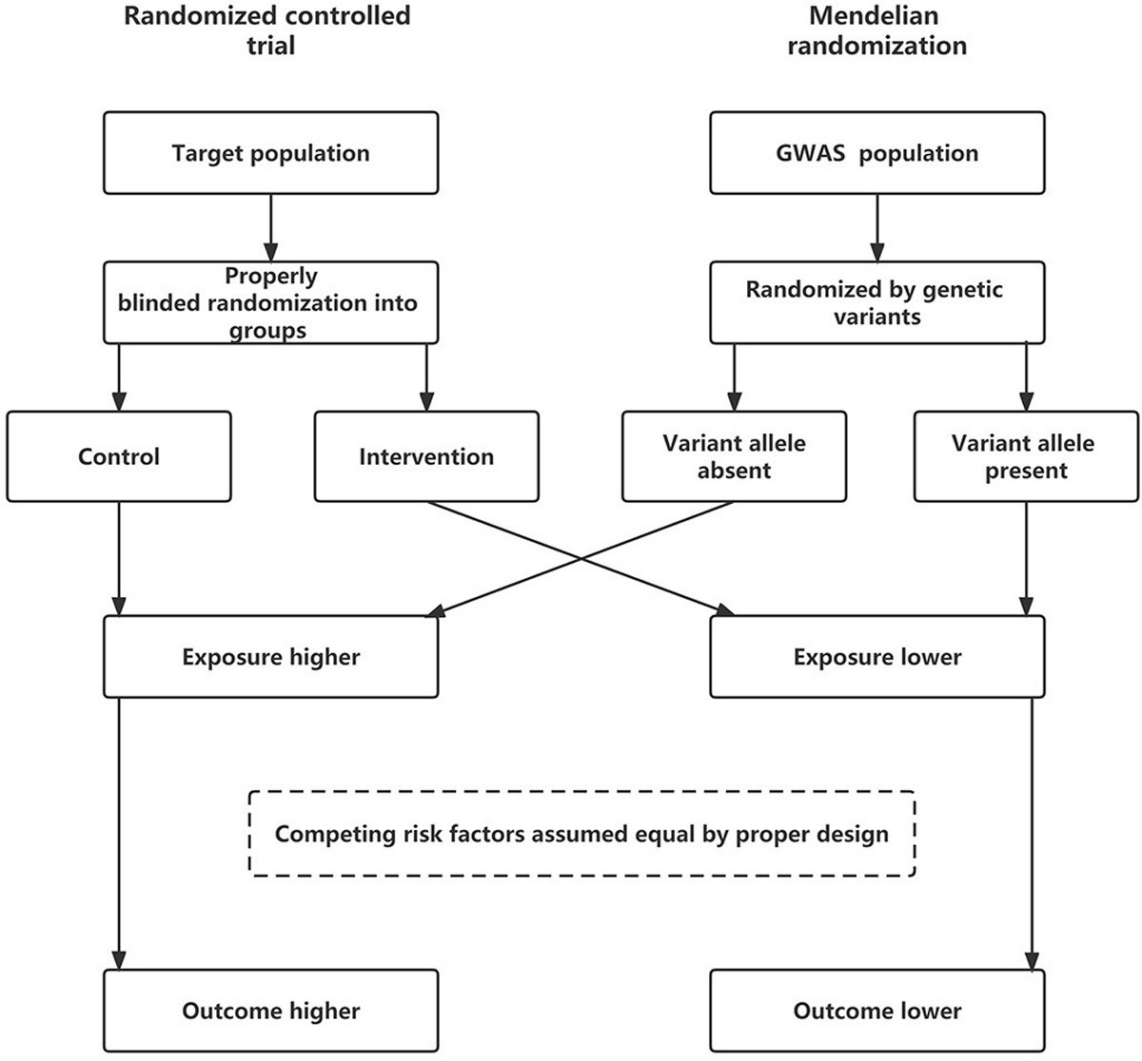
Inferences about the causal effect of an exposure on an outcome in MR and its comparison with RCT. The fundamental conditions for a genetic variant to be an IV are summarized as the following: i. the variant is associated with the exposure, ii. the variant is not associated with the outcome via a confounding pathway, and iii. the variant does not affect the outcome directly, only possibly indirectly via the exposure [12]. Assuming that the RCT is properly blinded and randomized and that the IVs for MR analysis are valid, subgroups should differ systematically in the exposure but not in any other factor except for those causally downstream of the exposure. Therefore, a difference in the average outcome between these subgroups would indicate a causal effect of the exposure on the outcome. Inferring a causal effect of the exposure on the outcome from an association between the IV and the outcome is analogous to inferring an intention-to-treat effect from an association between randomization and the outcome in an RCT [12, 13].

Mendelian randomization (MR) is a causality inference method that uses genetic variants in nonexperimental data as instrumental variables (IVs) to make inferences about the causal effect of an exposure on an outcome. With valid IVs selection, MR could reach a causal inference similar to that of a randomized control trial (RCT) without complete clarification of confounders [12–14] (Fig 1). Bidirectional MR (BIMR), two-step MR and multivariable MR (MVMR) are extensions of univariable MR (UVMR) that provide clearer causal estimates between the exposure and outcome [15–17].

To the best of our knowledge, the causality between SLE, T1DM and 25-OHD level has not been well assessed in epidemiological studies. We performed multiple MR analysis to explore the causal relationship between T1DM, 25-OHD level and SLE in the hope of shedding more light on the link between organ-specific and systematic autoimmunity, and the important role of 25-OHD in the prevention and cure of autoimmune disease.

## Materials and methods

In a two-sample MR framework, analysis were applied using summary-level genetic data from human genome-wide association studies (GWAS) based on the British UK Biobank [18].

### Genetic instrumental variables

Genetic IVs for T1DM were identified using results from the largest available GWAS for T1DM conducted by Onengut-Gumuscu et al. [19], which comprised 6683 cases and 22969 controls of European ancestry. Genetic IVs for SLE used in this study were extracted from the largest GWAS, which comprised 7219 cases and 15991 controls of European ancestry conducted by Bentham et al. [20]. We drew on summary statistics from the largest and most recent meta-analysis GWAS for the 25-OHD level (n = 441,291) conducted by Manousaki et al. [21]. As a continuous variable, we expressed our MR estimates as the causal effect of a given nmol/l increase in 25-OHD level (corresponding to 1 standard deviation in standardized natural log-transformed 25-OHD).

To ensure the validity of the IV assumption, IVs for MR analysis were selected based on the following criteria: (I) r^2^ measure of linkage disequilibrium among instruments <0.001 at a clumping distance cut-off of 10000 kb; (II) *P* value less than the genome-wide significance level identified in the corresponding GWAS (5 × 10^−8^ for SLE, T1DM and 25-OHD level, respectively); and (III) minor allele frequency (MAF) > 0.3.

### Primary Mendelian randomization analysis

**Fig 2:**
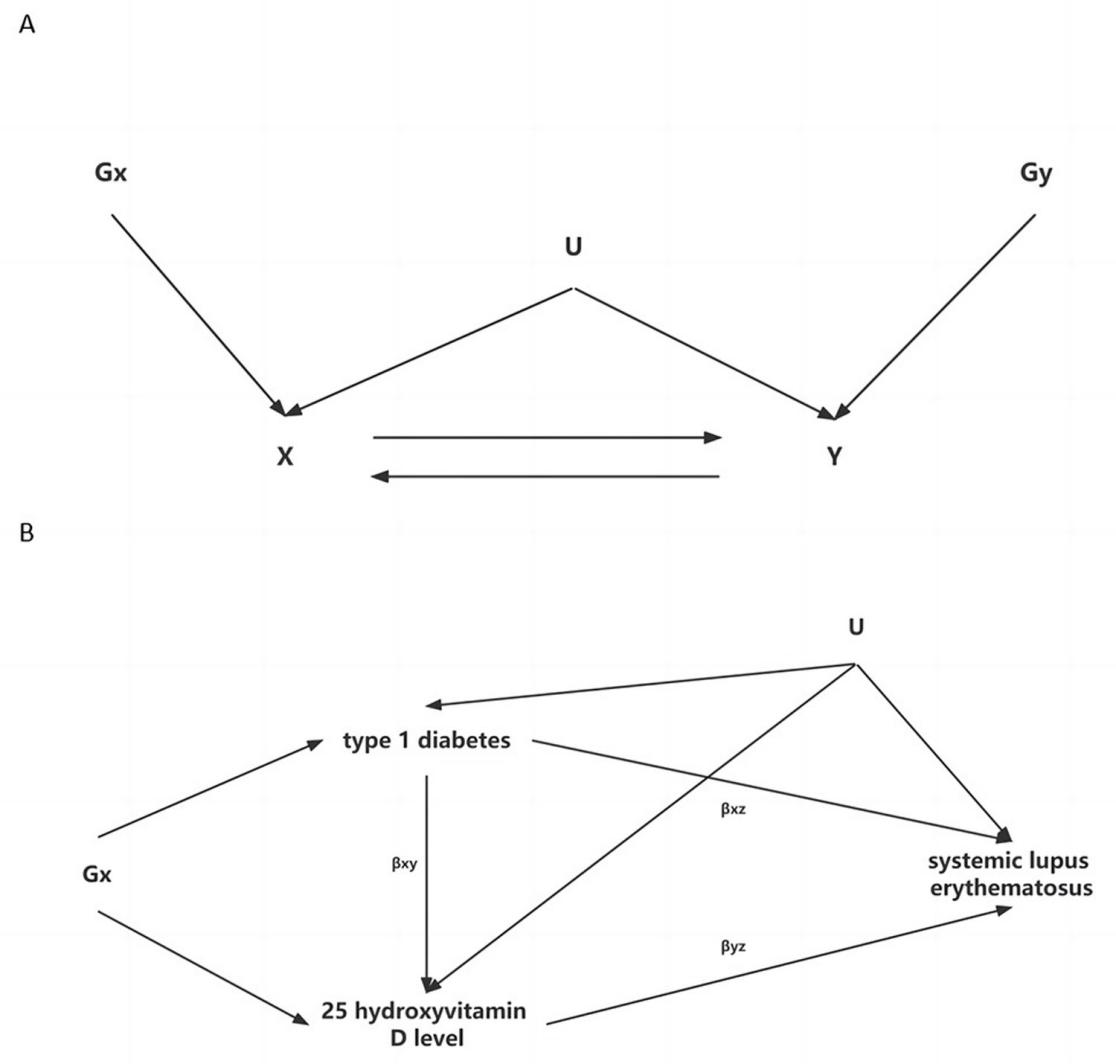
Directed acyclic graph of BIMR and MVMR. (A) BIMR: Gx can be used to estimate the causal effect of exposure X on outcome Y. Gy can be used to estimate the causal effect of outcome Y on exposure X. Genetic variants that are IVs for exposure X(Gx) and that are IVs for outcome Y(Gy) should be completely different. U indicates all confounders, which are assumed to be unknown. (B) MVMR: the total and direct effects of T1DM and 25-OHD level on SLE, based on the following IV assumptions for a genetic variant in MVMR: i. the variant is associated with one or more of the exposures, ii. the variant is not associated with the outcome via a confounding pathway, and iii. the variant does not affect the outcome directly, only possibly indirectly via one or more of the exposures. The direct effect of T1DM on SLE is the effect that T1DM has on SLE not via any other exposure variables, which is equal to βxz; similarly, the direct effect of the 25-OHD level on SLE is equal to βyz. The total effect of T1DM and 25-OHD level on SLE is the effect of T1DM on SLE directly plus the effect of T1DM on SLE via the 25-OHD level, which is equal to βxz + βxyβyz. U indicates all confounders, which are assumed to be unknown.

BIMR assesses the effect of the exposure on the outcome but also the effect in the opposite direction (Fig 2A). Specifically, on the condition that Gx and Gy were completely different, UVMR were first performed from T1DM to SLE, and then the direction was reversed. To evaluate the causal relationship between 25-OHD level and above diseases, BIMR was also conducted between 25-OHD level and SLE, and 25-OHD level and T1DM.

Based on the results of BIMR, we performed a two-step MR to explore whether 25-OHD level could serve as a mediator in the possible causal relationship of T1DM and SLE, and MVMR was conducted to investigate the direct causal effect of T1DM and 25-OHD level on SLE [16]. The total effect of T1DM and 25-OHD level on SLE is the effect of T1DM on SLE directly plus the effect of T1DM on SLE via the 25-OHD level, which is equal to βxz + βxyβyz (Fig 2B) [22]. The single nucleotide polymorphism (SNP) used to conduct MVMR were combinations of IVs that were associated with at least one exposure (genome-wide significant *P* value < 5 × 10^−8^) and were clumped on r^2^ < 0.001 within 10000 kb. We further split the IVs for MVMR analysis into those that only genome-wide significantly associated with T1DM (“T1DM-only SNP”), those that only genome-wide significantly associated with 25-OHD level (“25-OHD-only SNP”) and those that associated with both T1DM and 25-OHD level (“T1DM&25-OHD SNP”) by using the genome-wide significant *P* values. If the *P* value of both T1DM and 25-OHD level was less than 5 × 10^−8^, it was classified as the “T1DM&25-OHD SNP”.

### Sensitivity analysis

To ensure that the results of MR analysis were robust, several sensitivity analysis were performed. (I) To avoid weak IV bias, for BIMR, we calculated R^2^ (for each SNP), R^2^sum (for each exposure) and F-statistics to evaluate the strength of IVs; for MVMR, the conditional F-statistics were calculated [23]. (II) On the basis of the inverse-variance weighted (IVW) method [24], we continue to apply three robust analysis methods in BIMR to ensure that when some IVs cannot fully meet the IV assumptions, the results of MR analysis are still consistent: weighted median, weighted mode approaches and MR–Egger regression [25–27]. For MVMR, IVW method and MR-Egger regression were used [24, 27, 28]. The main results of MR analysis were described based on IVW method [24]. In addition, the MVMR-Lasso method was performed to evaluate the validity of SNPs to ensure that MR assumptions were not violated [29]. (III) Pleiotropy is an important reason for the violation of IV assumptions, which leads to inaccurate causal effects. The MR–Egger intercept test was used to evaluate horizontal and directional pleiotropy; the I_GX_^2^ statistic was used to check the violation of the no measurement error (NOME) assumption which states the potential relative bias due to measurement error [27, 28]. The Mendelian Randomization Pleiotropy Residual Sum and Outlier (MR-PRESSO) algorithm was used to identify IV outliers, and the MR-PRESSO global and MR-PRESSO distortion tests were used to evaluate horizontal pleiotropy [30]. (IV) The heterogeneity estimated by Cochran’s Q test and leave-one-out sensitivity analysis were used to assess whether any single IV was driving the results and to check for consistency of the analysis with MR assumptions for BIMR. For MVMR, Cochran’s Q statistics were used to assess IV strength heterogeneity [31].

All MR analysis were performed using the packages “Two Sample MR”, “Mendelian Randomization” and “MVMR” in R software (version 4.1.2).

### Ethics statement

Each GWAS included were permitted by their academic ethics review committees, and each participant signed written informed consent [19–21]. Since no primary data were used in this study, ethical approval was waived by the Ethics Committee of the First Hospital of Jilin University.

## Results

### Genetic instrumental variables

For BIMR, a total of 196 SNPs robustly and independently associated with the corresponding outcomes were included under the condition that the SNPs of BIMR in two opposite directions were completely different (S1 Table in S2 File). Outliers detected by MR-PRESSO algorithm were reported except for the UVMR analysis using 25-OHD level as the exposure and SLE as the outcome (S1 Table in S2 File). The F-statistic values for individual SNP ranged from 10 to 1193 (S1 Table in S2 File) and the F-statistic values for each exposure ranged from 12.04 to 65.18, which indicated that there was no evidence for weak IV bias (S2 Table in S2 File).

In MVMR analysis, 44 SNPs were identified as effective IVs, which included 26 IVs as “T1DM-Only SNP”, 11 IVs as “25OHD-Only SNP” and 7 IVs as “T1DM&25-OHD level SNP”. The MR-PRESSO algorithm did not detect outliers (S3 Table in S2 File).

### Bidirectional MR and two-step MR analysis

In the primary analysis of BIMR (S4 Table in S2 File and S1-S4 Figs in S1 File), we observed that T1DM increased the risk of SLE (OR_IVW_ = 1.192, 95% CI = 1.058–1.344, *P*_IVW_ = 4.05×10^−3^), and 25-OHD level was negatively associated with the risk of SLE (OR_IVW_ = 0.602, 95%CI = 0.431–0.840, *P*_IVW_ = 2.85×10^−3^). 25-OHD level had no causal effect on the risk of T1DM (OR_IVW_ = 0.275, 95% CI = 0.024–3.049, *P*_IVW_ = 0.293). In contrast, although the effect was weak, a negative causal effect of T1DM on 25-OHD level was observed (OR_IVW_ = 0.994, 95% CI = 0.989–0.999, *P*_IVW_ = 0.032). In BIMR analysis, there was no evidence to support causal associations of SLE on T1DM (OR_IVW_ = 1.053, 95% CI = 0.997–1.112, *P*_IVW_ = 6.16×10^−2^) and 25-OHD level (OR_IVW_ = 1.003, 95% CI = 0.995–1.007, *P*_IVW_ = 8.76×10^−2^). In the subsequent sensitivity analysis, the MR–Egger intercept, MR-PRESSO global test and MR-PRESSO distortion test suggested that IVs used in the primary MR analysis had evidence of pleiotropy and outliers (S1 and S5 Tables in S2 File). Meanwhile, Cochran’s Q test for heterogeneity showed that there was heterogeneity between some IVs in the MR-IVW and MR–Egger models (S6 Table in S2 File).

After the outliers in the primary BIMR analysis were eliminated by the MR-PRESSO algorithm, the BIMR results did not change significantly (Table 1 and S7 Table in S2 File and S5-S8 Figs in S1 File). We still observed an positive causality of T1DM on the risk of SLE (OR_IVW_ = 1.264, 95% CI = 1.150–1.389, *P*_IVW_ = 1.17×10^−6^), a negative causality of 25-OHD level on the risk of SLE (OR_IVW_ = 0.597, 95% CI = 0.427–0.838, *P*_IVW_ = 2.62×10^−3^) and a negative causal effect of T1DM on 25-OHD level (OR_IVW_ = 0.995, 95% CI = 0.991–0.999, *P*_IVW_ = 3.05×10^−2^). In the subsequent sensitivity analysis, the MR–Egger intercept, MR-PRESSO global test and MR-PRESSO distortion test did not suggest evidence of horizontal or directional pleiotropy (S5 Table in S2 File); Cochran’s Q test did not indicate heterogeneity among IVs, and I_GX_^2^ was more than 0.9 in all BIMR analysis, which suggests that NOME violation for IVs did not exist (S6 Table in S2 File).

**Table 1:** Causal relationship between SLE, T1DM and 25-OHD level estimated by BIMR.

Expousre	Outcome	*n*.SNPs	*P*ivw	OR (95%CI)
***BIMR of SLE AND T1DM***				
T1DM	SLE	25	1.17×10–6	1.264(1.150–1.389)
SLE	T1DM	12	0.069	1.042(0.996–1.089)
***BIMR of SLE AND 25-OHD level***				
25-OHD level*	SLE	69	2.62×10–3	0.597(0.427–0.835)
SLE	25-OHD level*	38	0.088	1.003(0.995–1.007)
***BIMR of T1DM AND 25-OHD level***				
T1DM	25-OHD level*	32	0.03	0.995(0.991–0.999)
25-OHD level*	T1DM	4	0.106	0.301(0.070–1.288)

Abbreviations: BIMR, bidirectional mendelian randomization; SLE, systemic lupus erythematosus; T1DM, type 1 diabetes; 25-OHD, 25 hydroxyvitamin D; n.SNPs, number of SNPs used in MR; IVW, inverse-variance weighted; OR, odds ratio.

*Each standard deviation increase of 25-OHD level

Based on the results of BIMR analysis and two-step MR analysis, there was strong evidence that T1DM could increase the risk of SLE while negatively associated with 25-OHD level, 25-OHD level had a negative causal relationship with the risk of SLE. Decreased 25-OHD level might be a mediator in the causal relationship between T1DM and SLE. Therefore, there was a possible causal network relationship between T1DM, 25-OHD level and SLE (Fig 2B). MVMR analysis was continuously used to verify the direct causality of T1DM and 25-OHD level on SLE.

### Multivariable MR analysis

Primary MVMR provided evidence that T1DM increased the risk of SLE (OR_MVMR-IVW_ = 1.149, 95% CI = 1.042–1.266, *P*_MVMR-IVW_ = 5.35×10^−3^, Table 2). However, although the direction of causal effects suggested by the scatter plot of individual SNP potential effects was consistent with that in BIMR analysis (S9 Fig in S1 File), no direct causal effect between 25-OHD level and risk of SLE was found (OR_MVMR-IVW_ = 0.360, 95% CI = 0.047–2.757, *P*_MVMR-IVW_ = 0.325, Table 2). Nevertheless, in the sensitivity analysis, Cochran’s Q-statistic for IV validity suggested strong heterogeneity among IVs (*P* = 2.35×10^−18^, Table 2), which suggested that primary MVMR analysis violated the exclusion restriction assessment in MR analysis, which led to the bias of causal effect inference [28]. In addition, although the MR-PRESSO algorithm did not detect significant outliers, the MR-PRESSO global test provided strong evidence of significant horizontal pleiotropy between SNPs (*P*<0.001, S5 Table in S2 File). Therefore, we applied the MVMR-Lasso method to detect invalid SNPs and conduct robust MVMR analysis [27].

**Table 2:** Causal relationship of T1DM and 25-OHD on SLE estimated by MVMR.

Expousre	*n*.SNPs	F stat	Method	OR(95%CI)	*P*	*Pinter* ^*#*^	Q stat	Q stat	*P* value
							for IV strength*	for IV validity	for IV validity**
***MVMR***									
T1DM	31	66.982	IVW	1.149 (1.042–1.266)	5.35×10–3	0.914	2947.19	174.24	2.35×10–18
			MR EGGER	1.153 (1.041–1.276)	0.006				
25-OHD level##	13	20.372	IVW	0.360 (0.047–2.757)	0.325		896.38		
			MR EGGER	0.416 (0.029–6.022)	0.521				
***MVMR-Lasso***									
T1DM	23	34.204	IVW	1.249 (1.148–1.360)	1.25×10–5	0.605	1162.95	30.42	0.496
			MR EGGER	1.251 (1.148–1.364)	0.001				
25-OHD level##	11	24.112	IVW	0.305 (0.109–0.857)	0.031		819.82		
			MR EGGER	0.244 (0.063–0.942)	0.041				

Abbreviations: MVMR, multivariable mendelian randomization; SLE, systemic lupus erythematosus; T1DM, type 1 diabetes; 25-OHD, 25 hydroxyvitamin D; n.SNPs, number of SNPs used in MR; IV, instrumental variables; IVW inverse variance weighted; OR, odds ratio; F stat, conditional F-statistic; Q stat, Cochran’s Q statistics.

* Cochran’s Q statistics for assessing instrument strength, a value equal to or greater than 10 can be used as a minimum threshold for instrument strength.

** A p-value corresponding to the heterogeneity measure for instrument validity, observed heterogeneity is indicative of a violation of the exclusion restriction assumption in MR, which can result in biased effect estimates.

# The p-value for the MR-Egger intercept test (a low p-value suggests either directional pleiotropy or failure of the Instrument Strength Independent of Direct Effect assumption, and its significance indicates that the IVW estimate is biased).

## Each standard deviation increase of 25-OHD level.

**Fig 3:**
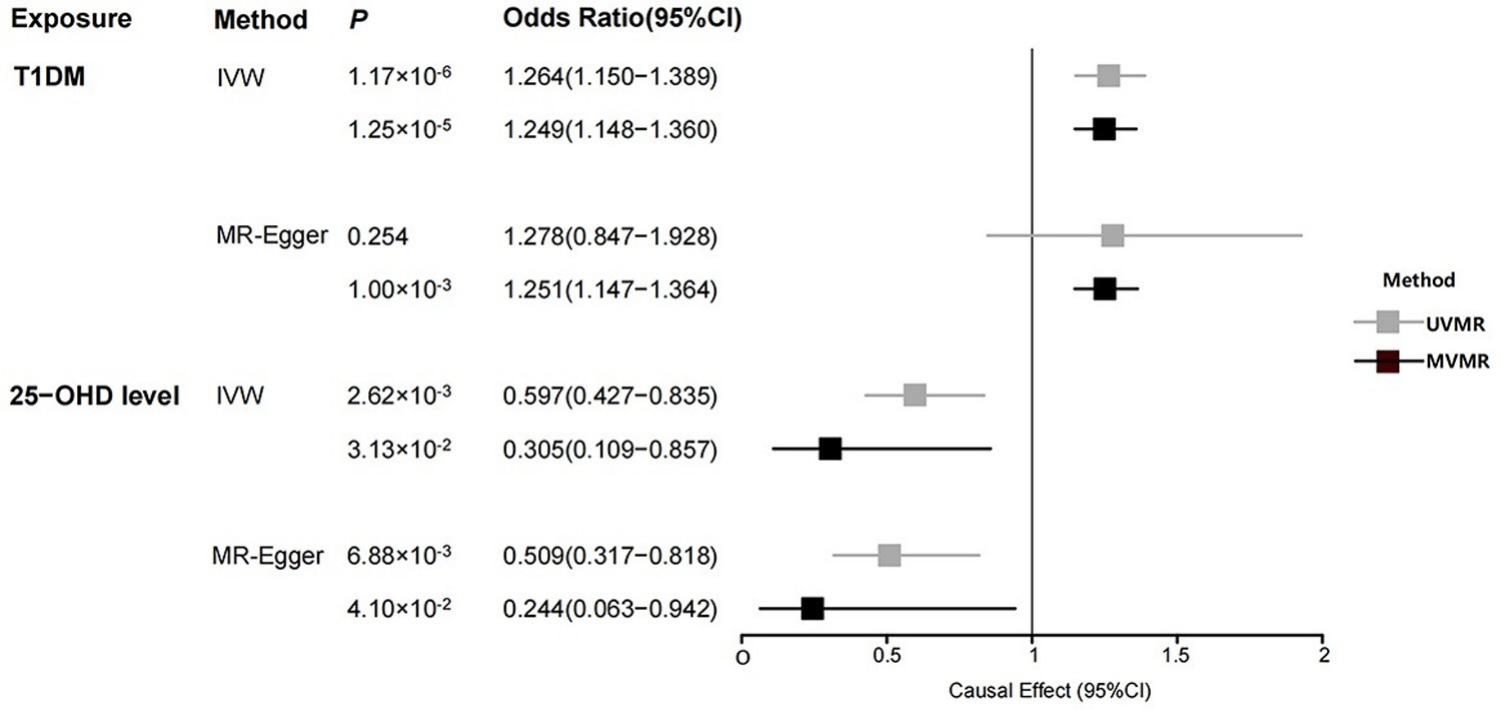
Odds ratios and 95% confidence intervals for the causal effect of T1DM and 25-OHD level on SLE in UVMR and MVMR-Lasso analyses. The colours of the fitted lines indicate two MR analyses. UVMR, univariable Mendelian randomization; MVMR, multivariable Mendelian randomization; SLE, systemic lupus erythematosus; T1DM, type 1 diabetes; 25-OHD, 25 hydroxyvitamin D; N. SNPs, number of SNPs used in MR; IV, instrumental variables; IVW inverse variance weighted.

After eliminating 10 invalid SNPs (Table 2 and S10 Fig in S1 File), MVMR analysis indicated a direct causal effect of T1DM on the risk of SLE (OR_MVMR-IVW_ = 1.249, 95% CI = 1.148–1.360, *P*_MVMR-IVW_ = 1.25×10^−5^), and 25-OHD level was negatively associated with the risk of SLE (OR_MVMR-IVW_ = 0.305, 95% CI = 0.109–0.857, *P*_MVMR-IVW_ = 0.031), which remained consistent with the BIMR analysis (Fig 3). Neither the conditional F-statistic nor Cochran’s Q-stat for IV strength suggests the existence of weak IV bias (Table 2). In the sensitivity analysis, MR-PRESSO global test (*P* = 0.446, S5 Table in S2 File) and MR-Egger intercept test (*P*_inter_ = 0.605, Table 2) did not find evidence of horizontal pleiotropy between SNPs. Cochran’s Q-stat for IV validity did not prompt the heterogeneity between IVs, and the results of the MVMR-Lasso method did not violate the exclusion restriction assessment in MR analysis (Table 2). Therefore, we suppose the results of the MVMR-Lasso method were robust, reliable and consistent with the BIMR results. The network causal relationship between 25-OHD level, T1DM and SLE was verified by MVMR (Fig 2B).

## Discussion

In this study, we applied BIMR, two-step MR and MVMR analysis to determine a network causal relationship between T1DM, 25-OHD level and SLE, the causality between different types of autoimmunity and the role of environmental triggers in the pathogenesis of different types of autoimmunity has been discussed.

Supporting our findings regarding an independent effect of T1DM on SLE, an association between these two diseases has been found in cohort studies and case reports, with a relatively high prevalence of anti-insulin antibodies in patients with SLE [2, 32, 33]. Through MR analysis, a positive causal effect of T1DM on the risk of SLE has been observed without reverse causality. The immunogenetic mechanisms of this causality present an intriguing unresolved problem of autoimmune predisposition. Some scholars have proposed a possible hypothesis: in multiple autoimmunity, two discrete epitopes originating from the same autoantigen may interact with each of the human leukocyte antigen (HLA) specificities, eliciting the production of both types of autoantibodies [34]. Another hypothesis may reveal how different autoantigens lead to multiple autoimmunity in the same patient: each of the disease-specific autoantigens may bind to either HLA protein, leading to the induction of both diseases by cross-presentation [34, 35]. In the study of Kottyan LC et al., it was pointed out that some shared non-HLA local contributions to variation in the immune system that alters the presentation of the driving autoimmune diseases to include alternative moieties (e.g., *DOCK3*, *C4A*, *BLK*, *ERI1*) [36]. Meanwhile, other MR analysis also prompted the causal relationship between organ-specific autoimmunity and SLE; for instance, celiac disease and HT might increase the risk of SLE [37, 38]. Combining with our results, MR analysis provided experimental exploration direction for verifying relevant hypotheses and helped to further clarify the complex pathogenesis of autoimmune diseases.

25-OHD deficiency was considered as one of the environmental triggers of SLE, as a sterol hormone, 25-OHD can exert immunoregulatory effects on the proliferation, differentiation and function of activated immune cells through VDR on the surface of immune cells [8, 39, 40]. Specifically, APCs became more immature and tolerogenic by decreasing antigen presentation and increasing the expression of inhibitory receptors on the cell surface [8]. For activated B cells, 25-OHD could inhibit their differentiation into memory B cells and plasma cells; induce apoptosis; and increase the expression of anti-inflammatory cytokines and receptors, such as interleukin-10 and CC chemokines receptors10 [39, 41]. The activation of APCs and B cells played an important role in the pathogenesis of SLE [3], in vitro, the abnormal autoimmune response could also be inhibited by supplementation with exogenous 25-OHD, leading to a reduction in the production of autoantibodies [42]. Consistent with our analysis, 25-OHD level was negatively associated with the risk of SLE, observational studies also showed that the serum 25-OHD level of SLE patients is significantly lower than that of healthy people [10, 39]. Moreover, the latest RCT also demonstrated that exogenous 25-OHD supplementation could prevent a variety of systemic autoimmune diseases, including rheumatoid arthritis and SLE, despite administration with or without omega 3 fatty acids [43]. Therefore, we speculated that the insufficient inhibition of 25-OHD signaling pathway on activated immune cells due to the 25-OHD deficiency may further promote the occurrence of autoimmune inflammation in SLE.

The network causal relationship between T1DM, 25-OHD level, and SLE is enlightening for further understanding the pathogenesis of SLE. In the pathogenesis of SLE, the pathogenic role of environmental triggers was crucial [3]. Savastio S et al. reported that changes in insulin sensitivity and abnormal glycometabolism caused by T1DM can significantly decrease 25-OHD levels, which may be related to decreased 25-OHD synthesis in renal tubular epithelial cells caused by hyperglycemia [44]. Meanwhile, the imbalanced diet in T1DM patients may also contributed to 25-OHD deficiency [45]. The abnormal glycometabolism caused by T1DM can lead to a decrease in lymphocyte activation threshold, increasing the efficiency of autoantigens to activate lymphocytes, and promoting the onset of SLE; Yin Y et al. reported that hyperglycemia could induce the upregulation of glycolysis and mitochondrial oxidative metabolism in CD4+T cells, leading to an increase in immune response levels [46]. On the other hand, 25-OHD deficiency resulted in abnormal activated lymphocytes cannot be suppressed by immunoregulatory mechanisms, and also promoted the autoimmune inflammation in SLE [3, 39, 40]. In addition, genetic factors may also be involved in the pathogenesis of T1DM and SLE. In summary data-based MR analysis, it was reported that *BLK* gene mutation is causally associated with the onset of SLE [47], meanwhile, *BLK* gene mutation could be caused by maturity-onset diabetes of the young, including T1DM [48]. Further exploration of *BLK* gene function may contribute to know the comorbidity mechanism between T1DM and SLE. Consistent with the current consensus, the onset of SLE was the result of a combination of genetic susceptibility factors and environmental triggers [3], based on our findings, we speculated that abnormal glycometabolism caused by T1DM was a contributing factor to 25-OHD deficiency and SLE inflammation, the immunoregulatory dysfunction caused by 25-OHD deficiency was also a cause in the onset of SLE, and there were also important genetic susceptibility factors involved. We supposed that even in the pathogenesis of organ-specific autoimmunity and systemic autoimmunity comorbidities, the combined effect of environmental and genetic factors was the core link. More specifically, the corresponding endocrine and metabolic changes caused by an autoimmune disease may serve as a trigger or aggravating factor for another autoimmune inflammation, leading to another autoimmune disease, which could also be seen in the association between HT and SLE [1, 38].

Although the MR analysis has unique advantages in using non-experimental data for causal inference of exposure to outcomes, we should remain prudent about our findings due to the following limitations: (I) https://fanyi.baidu.com/?aldtype=16047-## When both exposure and outcome are binary variables, causal inference should be evaluated more carefully because the Wald-type estimation in the IVW method will lead to the bias of causal odds ratio [20]. Nevertheless, due to the validity of IVs and the consistency of causal inference by multiple approaches, we considered the impact is small. (II) Although we extracted genetic variation from GWASs with the largest sample size for MR analysis, a relatively small sample size led to a relatively conservative MAF value (MAF = 0.3) which may cause a decline in the reliability of causal inference. GWAS research with a larger sample size and more populations will improve the statistical power and extrapolation of MR analysis and obtain more robust and reliable causal inference results. (III) As only individuals of European ancestry were included in the GWASs from which we selected our IVs, the generalization of our findings to other populations was limited.

Taken together, we demonstrated that T1DM and 25-OHD level both have causal associations with the risk of SLE and that 25-OHD level could also be a mediator in the causality of T1DM and SLE. Further studies are needed to reveal the pathogenic mechanism and relationship between organ-specific autoimmunity and systematic autoimmunity. The role of environmental triggers related to immunoregulatory mechanisms in the pathogenesis of multiple autoimmunity is also worth further exploration.

## Supporting information

S1 File S1-S10 Figs are included in the file. (ZIP)

S2 File S1-S7 Tables are included in the file. (ZIP)
