# Supplementary material for: A network causal relationship between type-1 diabetes mellitus, 25-hydroxyvitamin D level and systemic lupus erythematosus: Mendelian randomization study
Source: PLoS One. 2023 May 17;18(5):e0285915. doi: 10.1371/journal.pone.0285915 (PMC10191345; doi:10.1371/journal.pone.0285915)
Supplement: S1 File — (ZIP) [file pone.0285915.s001.zip › S1 File.docx]

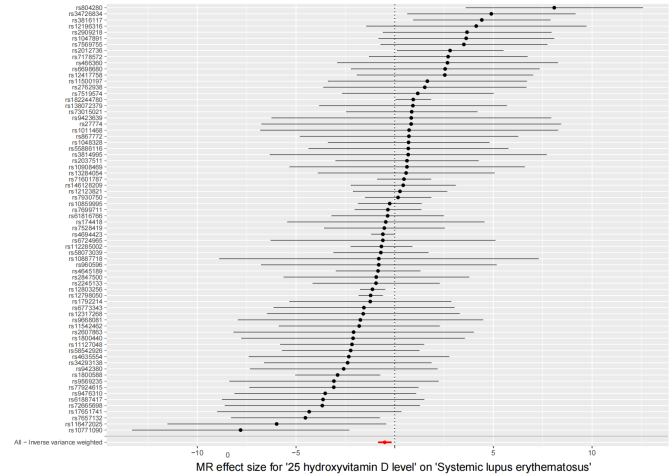


C


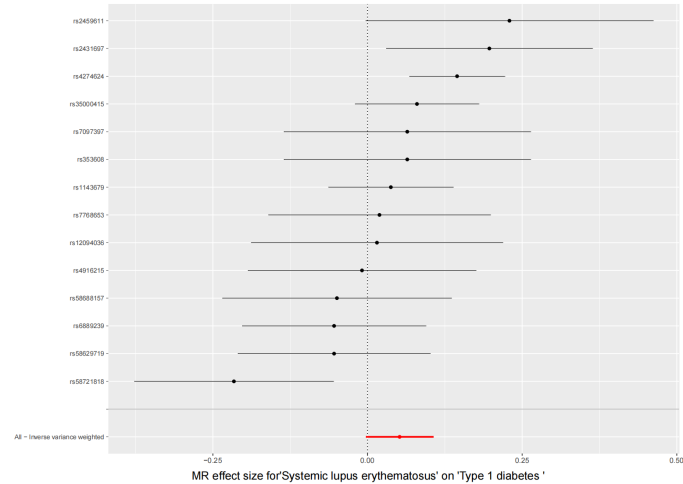


B


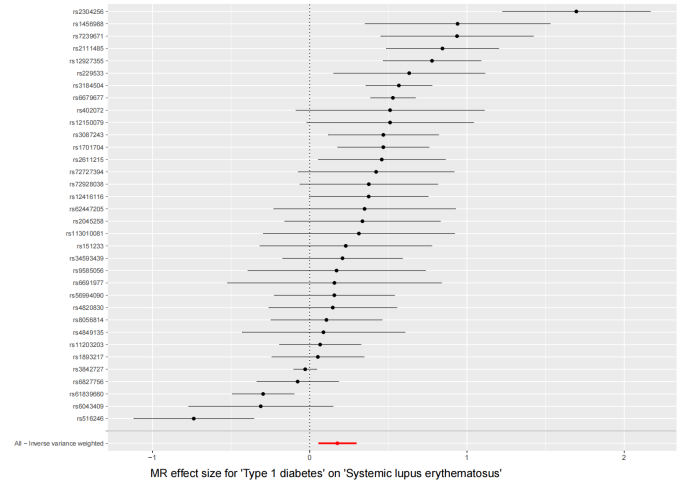


A


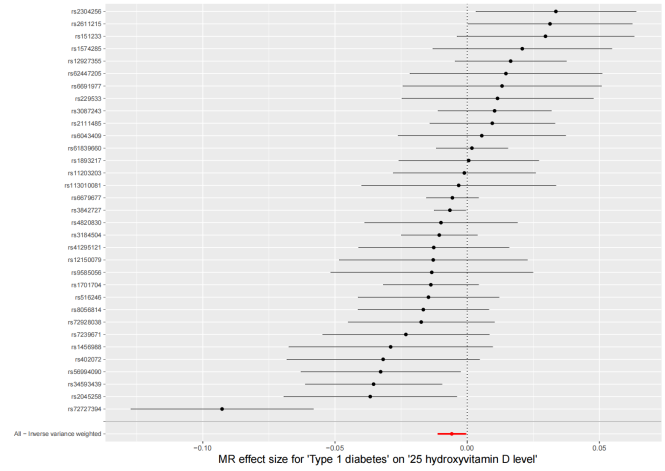


E

F


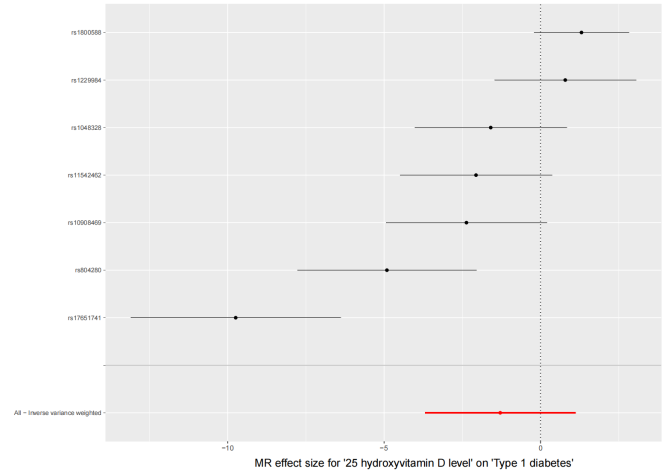

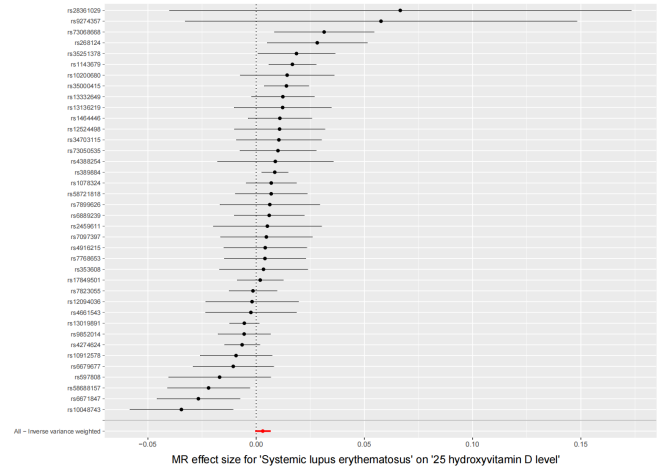


D

**S1 Fig. Forest plot of the causal effects of BIMR.** The causal effect of exposure on outcome was estimated using each SNP singly using the Wald ratio, and represented in a forest plot. (A-B) Forest plot of the BIMR analysis between T1DM and SLE. (C-D) Forest plot of the BIMR analysis between 25-OHD level and SLE. (E-F) Forest plot of the BIMR analysis between T1DM and 25-OHD level. BIMR, bidirectional mendelian randomization; SLE, systemic lupus erythematosus; T1DM, type 1 diabetes; 25-OHD, 25 hydroxyvitamin D.


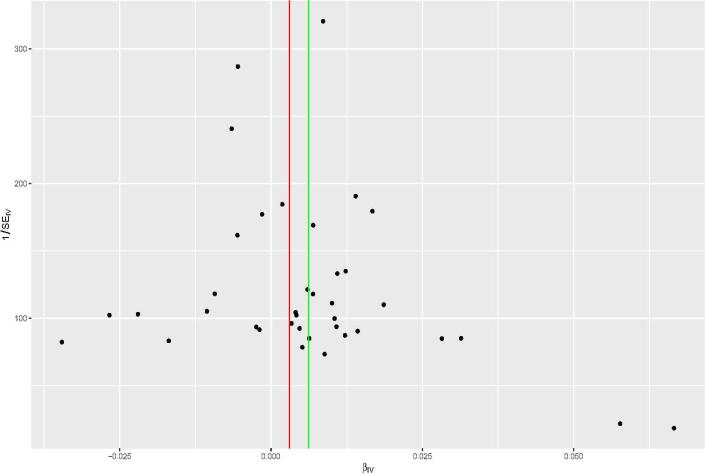


D


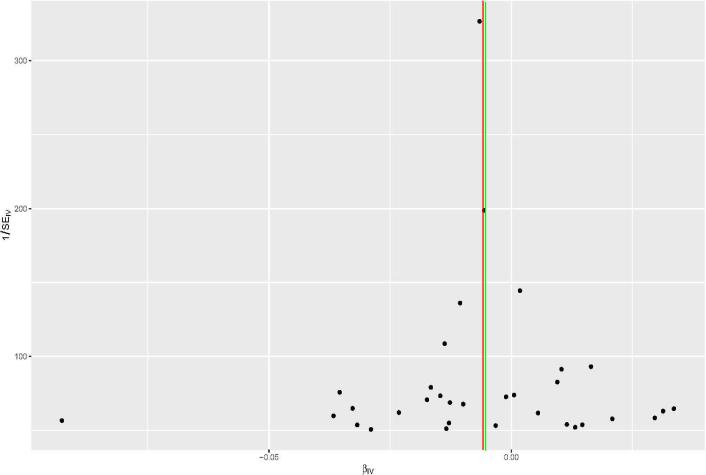


E

F


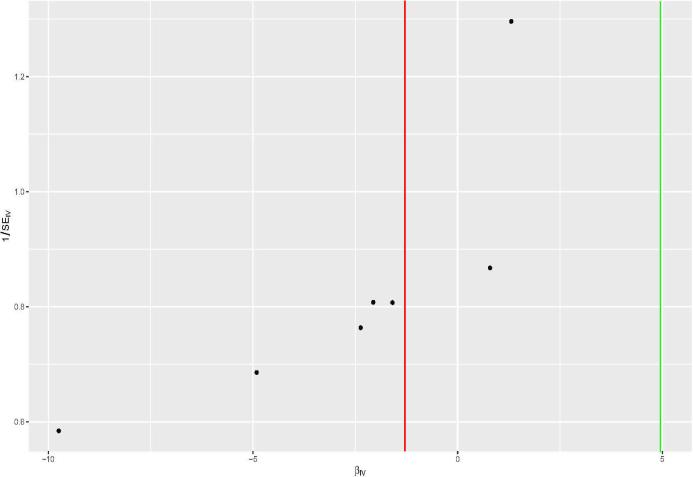


F

D


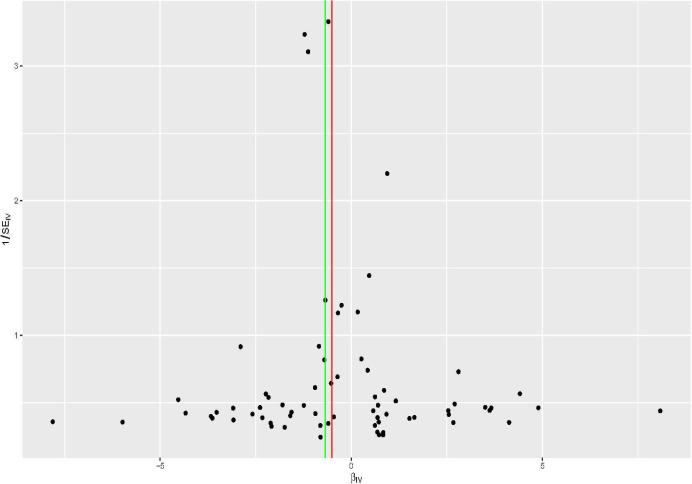


C

A


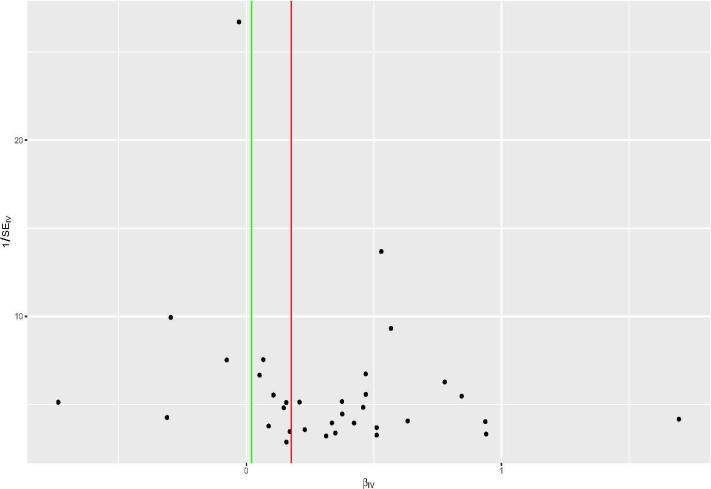

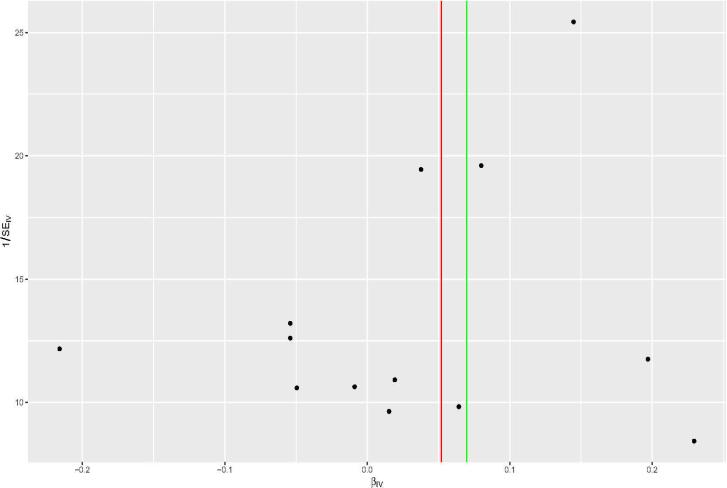


B

**MR-Egger**

**inverse variance weighted**


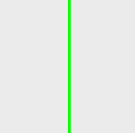

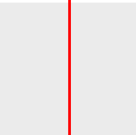


**S2 Fig. Funnel plots to show symmetrical distribution of individual variant estimates around the point estimate in BIMR.** The X axis represents the MR estimate of individual variants; the Y-axis represents the inverse of their standard error. Colors indicate for approaches used in univariable MR analyses. (A) Funnel plot of the MR analysis of T1DM-associated SNPs on SLE. (B) Funnel plot of the MR analysis of SLE-associated SNPs on T1DM. (C) Funnel plot of the MR analysis of 25-OHD level-associated SNPs on SLE. (D) Funnel plot of the MR analysis of SLE-associated SNPs on 25-OHD level. (E) Funnel plot of the MR analysis of T1DM-associated SNPs on 25-OHD level. (F) Funnel plot of the MR analysis of 25-OHD level-associated SNPs on T1DM.


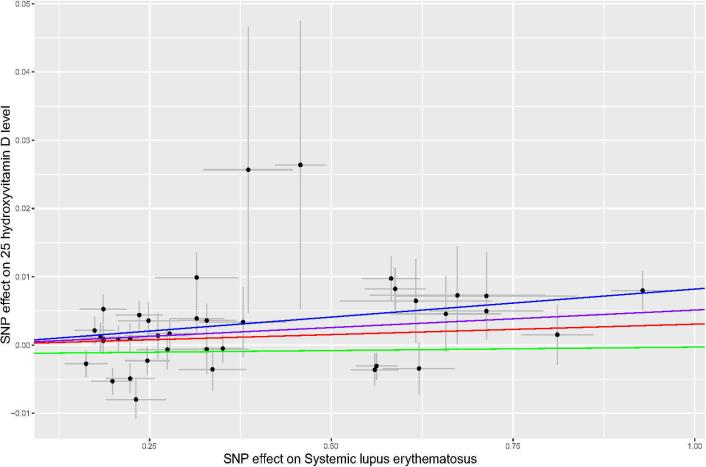


D


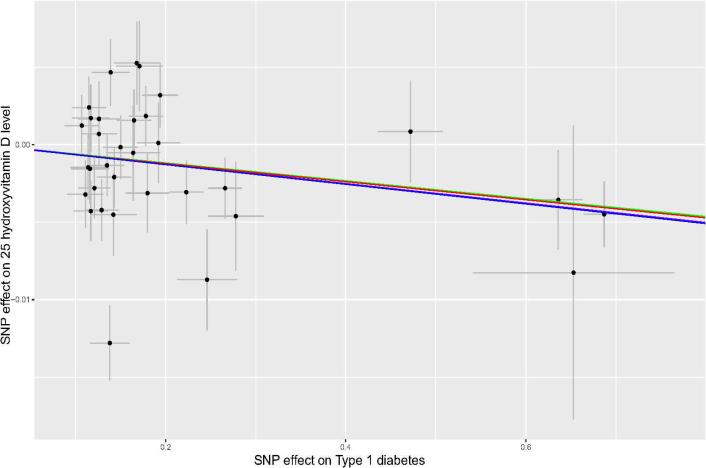


E

F


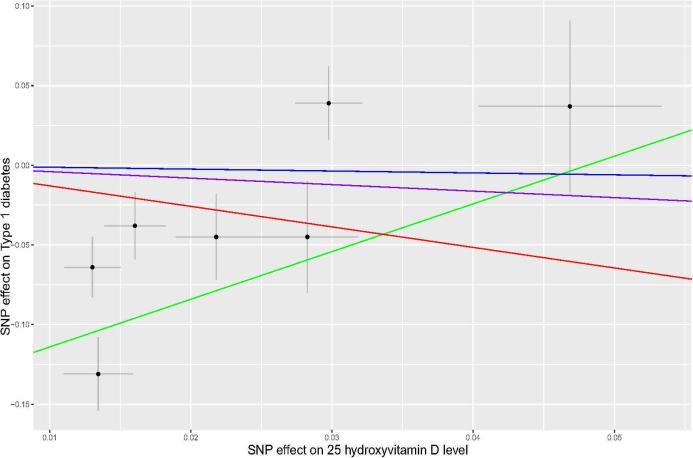

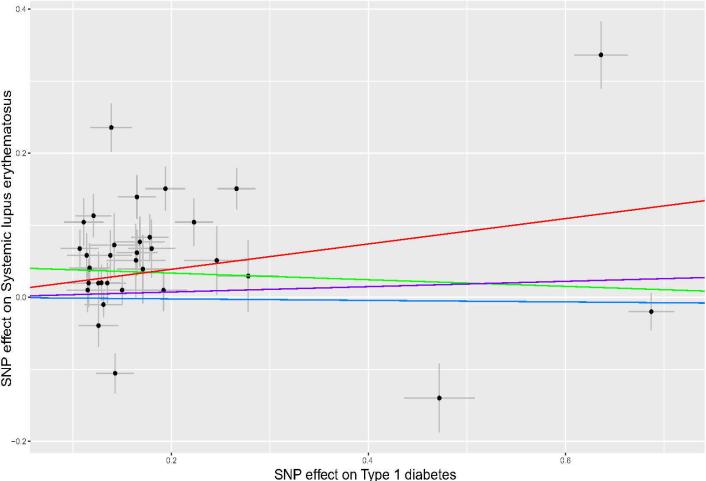


A


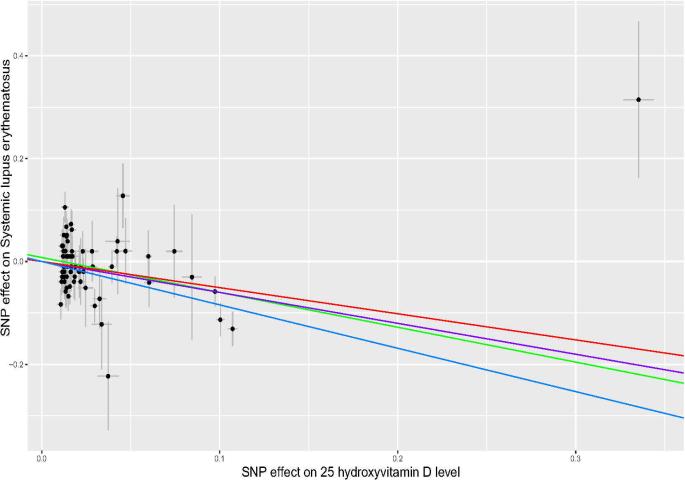


C


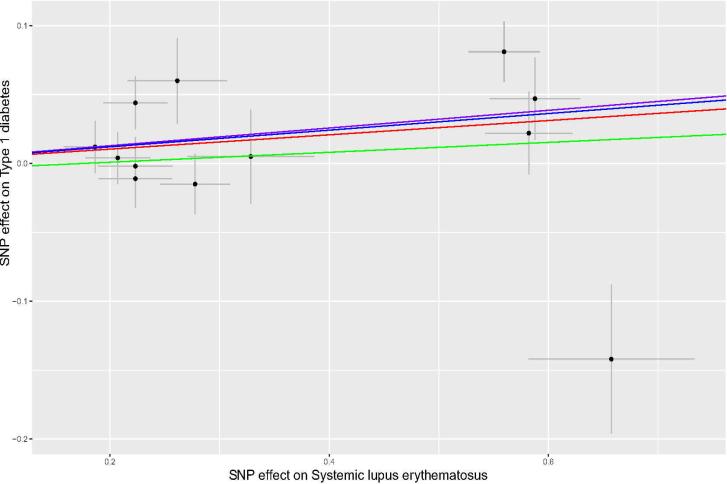


B

**MR-Egger**

**IVW**


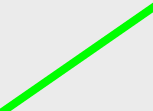

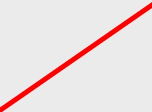


**Weighted mode**

**Weighted median**


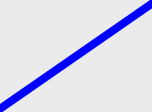

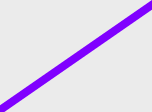


**S3 Fig. Scatter plot of SNP potential effects in BIMR analysis, with the slope of each line corresponding to estimated MR effect per method.** The X-axis represents the effect size of SNPs on exposure respectively; the Y-axis represents the effect size of SNPs on outcomes respectively. Colors of fitted line indicate for four approaches used in univariable MR analyses. (A-B) Scatter plot of the BIMR analysis between T1DM and SLE. (C-D) Scatter plot of the BIMR analysis between 25-OHD level and SLE. (E-F) Scatter plot of the BIMR analysis between T1DM and 25-OHD level. BIMR, bidirectional mendelian randomization; SLE, systemic lupus erythematosus; T1DM, type 1 diabetes; 25-OHD, 25 hydroxyvitamin D.


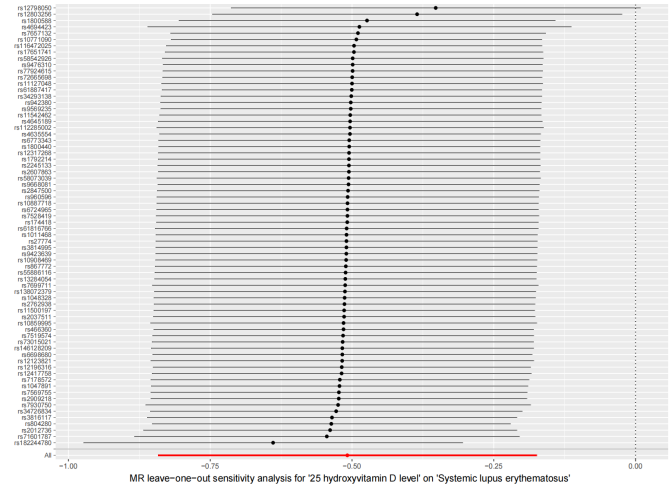


C


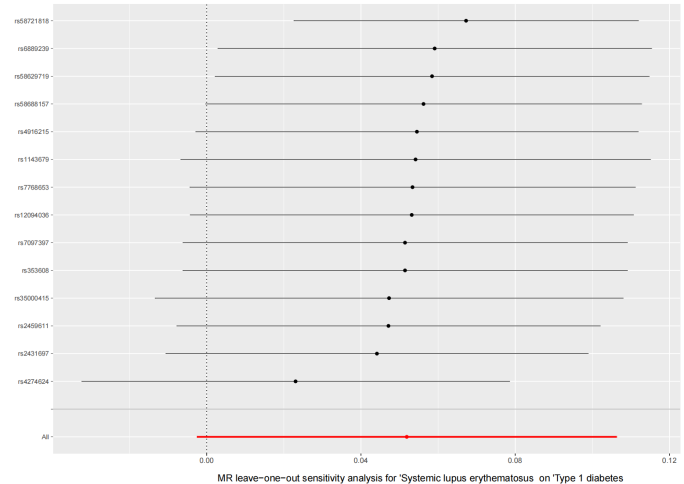


B


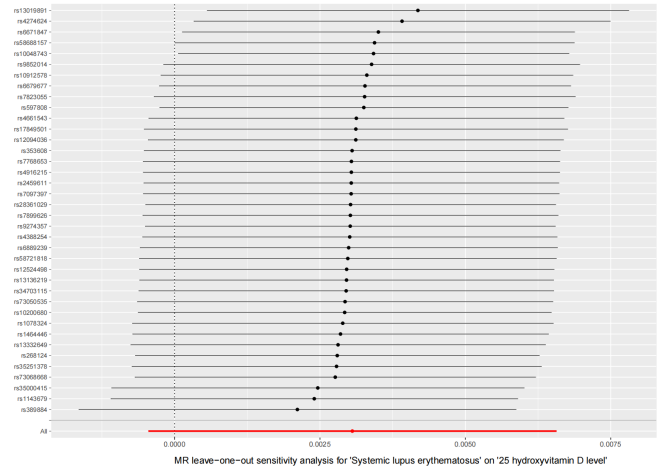


D


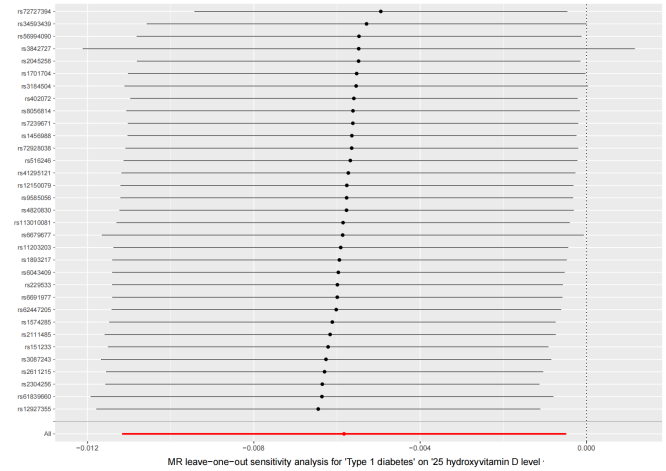


E

F


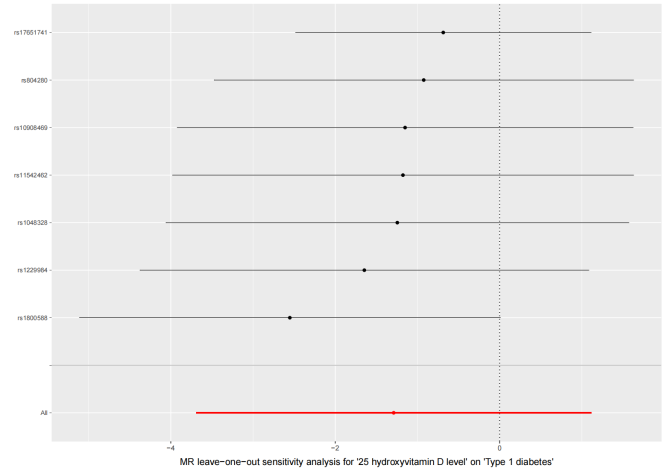

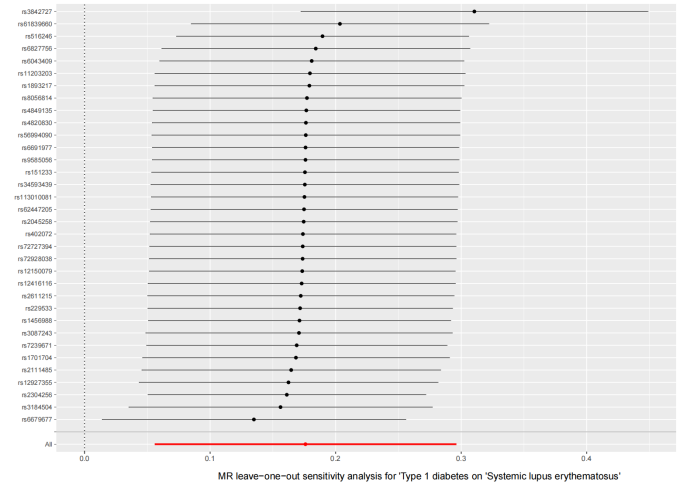


A


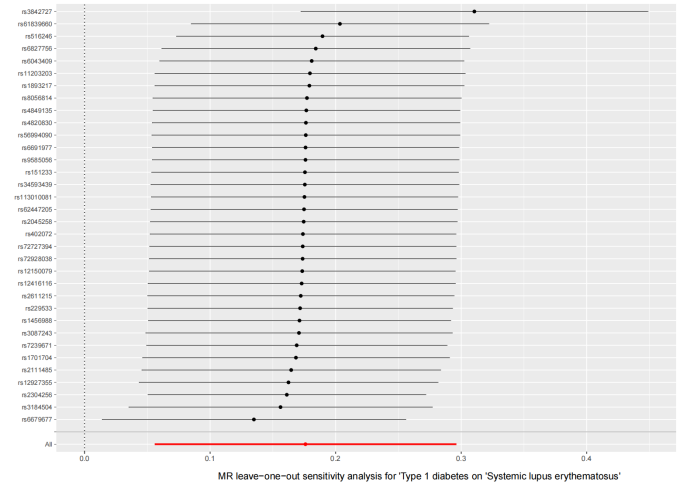


A

**S4 Fig. Leave-one-out sensitivity analysis for BIMR, a forest plot of causal estimates from the MR-IVW approach omitting each genetic variant in turn.** (A-B) Leave-one-out sensitivity analysis of the BIMR analysis between T1DM and SLE. (C-D) Leave-one-out sensitivity analysis of the BIMR analysis between 25-OHD level and SLE. (E-F)Leave-one-out sensitivity analysis of the BIMR analysis between T1DM and 25-OHD level. BIMR, bidirectional mendelian randomization; SLE, systemic lupus erythematosus; T1DM, type 1 diabetes; 25-OHD, 25 hydroxyvitamin D.


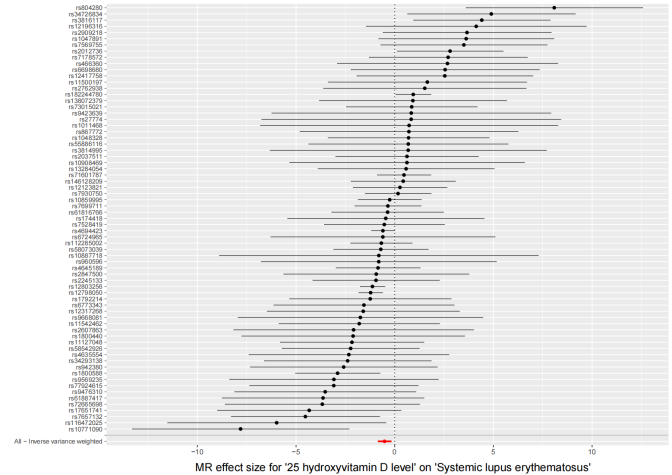


C*


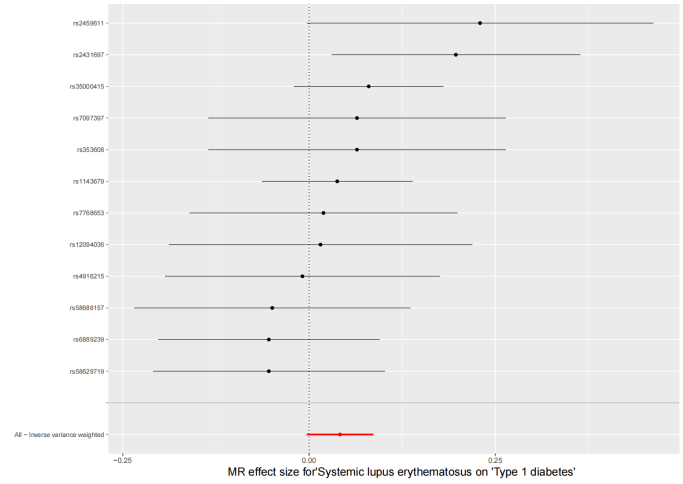


B


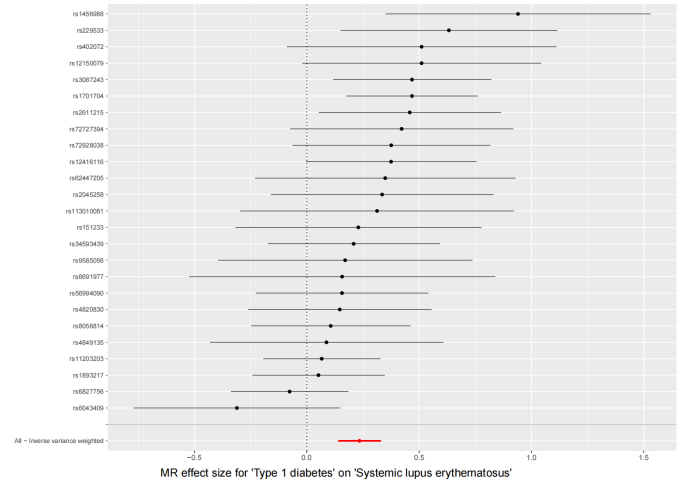


A


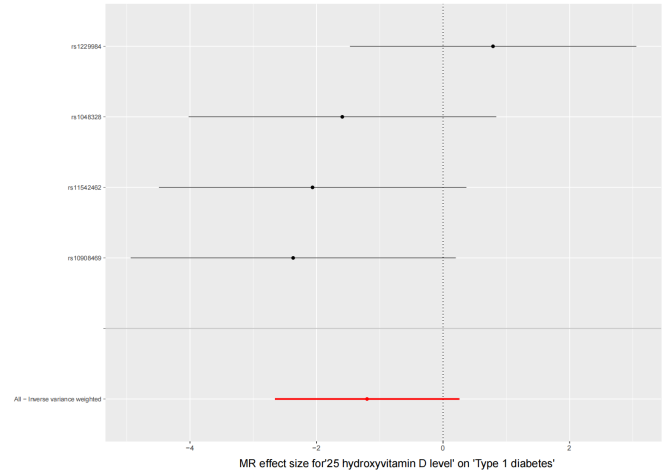


E


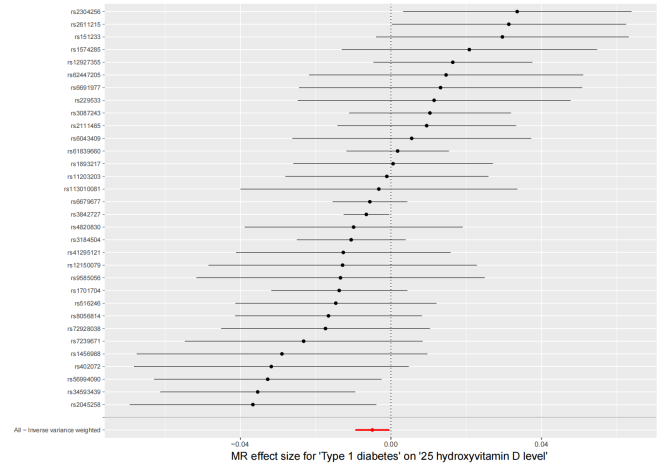


D

**S5 Fig. Forest plot of the causal effects of BIMR without outliers.** The causal effect of exposure on outcome was estimated using each SNP singly using the Wald ratio, and represented in a forest plot. (A-B) Forest plot of the BIMR analysis between T1DM and SLE without outliers. (C) Forest plot of the MR analysis of 25-OHD level-associated SNPs on SLE without outliers. (D-E) Forest plot of the BIMR analysis between T1DM and 25-OHD level without outliers. BIMR, bidirectional mendelian randomization; N.SNP is the number of genetic variants. SLE, systemic lupus erythematosus; T1DM, type 1 diabetes; 25-OHD, 25 hydroxyvitamin D.

*In MR analysis with SLE as exposure and 25-OHD level as result, there were no significant outliers identified by the MR-PRESSO test.


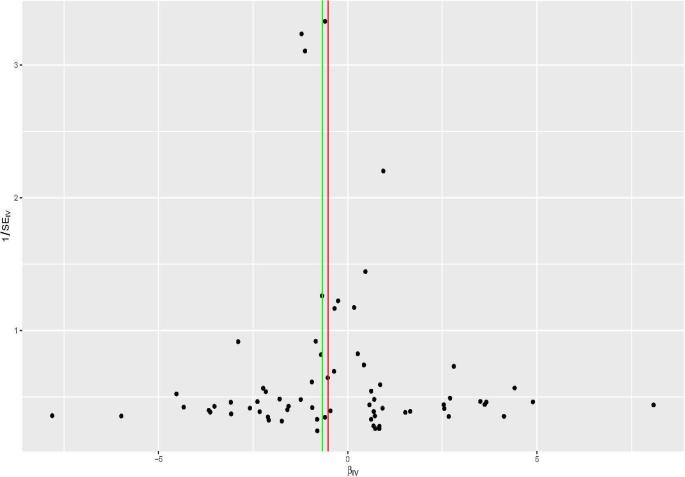


C*


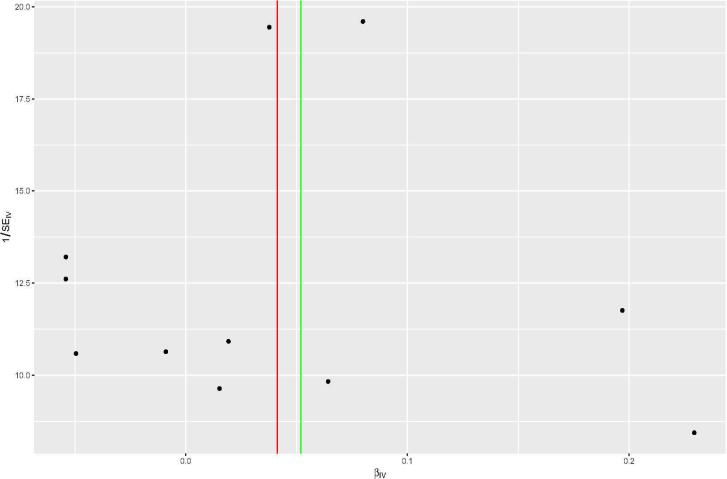


B


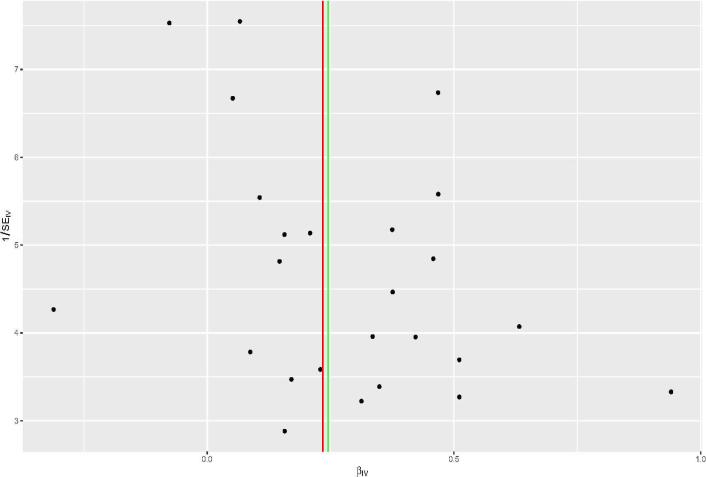


A


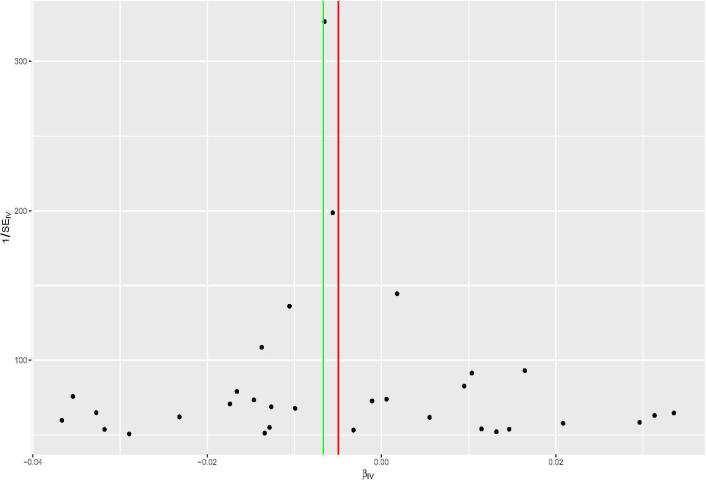


D


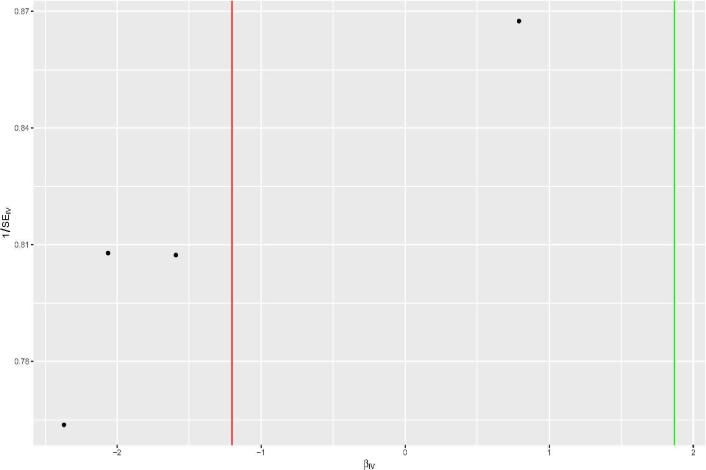


E

**MR-Egger**

**inverse variance weighted**


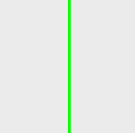

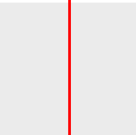


**S6 Fig. Funnel plots to show symmetrical distribution of individual variant estimates around the point estimate in BIMR without outliers.** The X axis represents the MR estimate of individual variants; the Y-axis represents the inverse of their standard error. Colors indicate for approaches used in univariable MR analyses. (A) Funnel plot of the MR analysis of T1DM-associated SNPs on SLE. (B) Funnel plot of the MR analysis of SLE-associated SNPs on T1DM. (C) Funnel plot of the MR analysis of 25-OHD level-associated SNPs on SLE. (D) Funnel plot of the MR analysis of T1DM-associated SNPs on 25-OHD level. (E) Funnel plot of the MR analysis of 25-OHD level-associated SNPs on T1DM.

*In MR analysis with SLE as exposure and 25-OHD level as result, there were no significant outliers identified by the MR-PRESSO test.


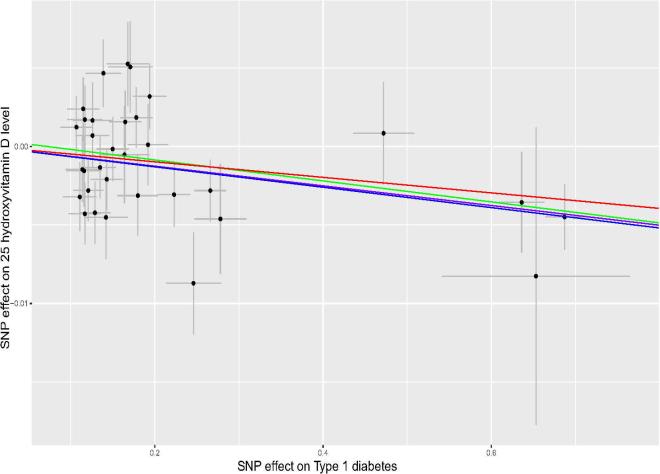


D


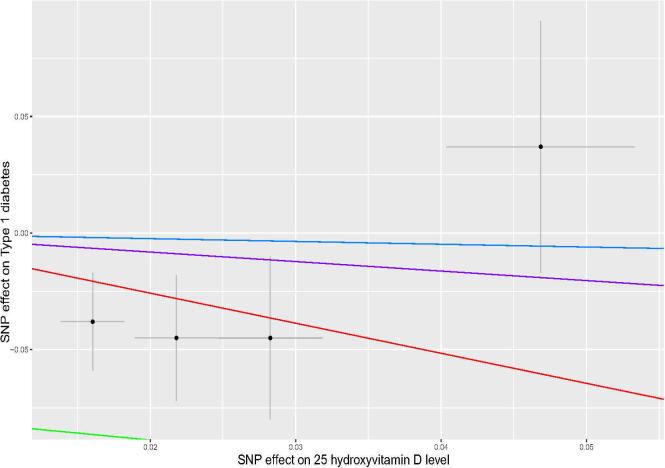


E


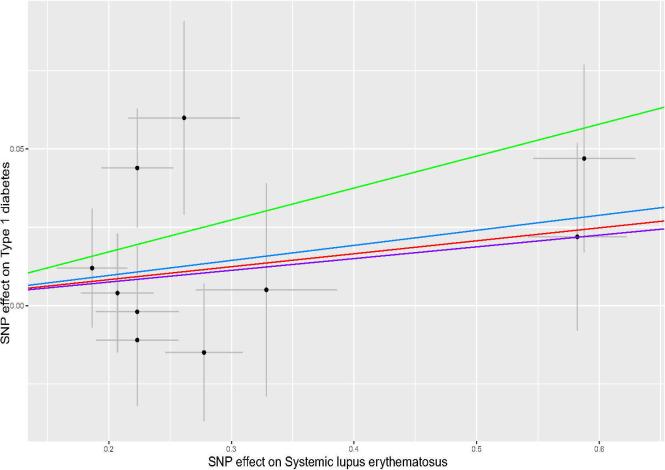

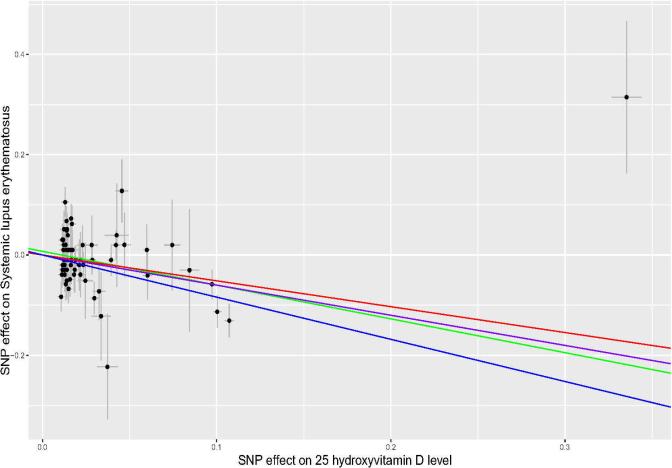


C

B


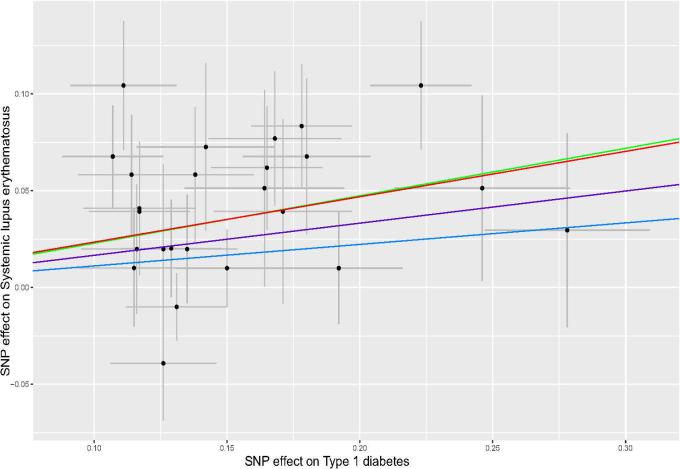


A

**MR-Egger**

**IVW**


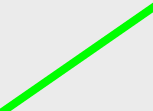

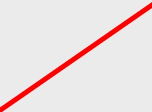


**Weighted mode**

**Weighted median**


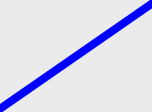

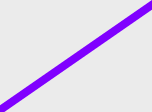


**S7 Fig. Scatter plot of SNP potential effects in BIMR analysis without outliers, with the slope of each line corresponding to estimated MR effect per method.** The X-axis represents the effect size of SNPs on exposure respectively; the Y-axis represents the effect size of SNPs on outcomes respectively. Colors of fitted line indicate for four approaches used in univariable MR analyses. (A) Scatter plot of the MR analysis of T1DM-associated SNPs on SLE without outliers. (B) Scatter plot of the MR analysis of SLE-associated SNPs on T1DM without outliers. (C) Scatter plot of the MR analysis of 25-OHD level-associated SNPs on SLE without outlier. (D) Scatter plot of the MR analysis of T1DM-associated SNPs on 25-OHD level without outlier. (E) Scatter plot of the MR analysis of 25-OHD level-associated SNPs on T1DM without outliers BIMR, bidirectional mendelian randomization; SLE, systemic lupus erythematosus; T1DM, type 1 diabetes; 25-OHD, 25 hydroxyvitamin D.

*In MR analysis with SLE as exposure and 25-OHD level as result, there were no significant outliers identified by the MR-PRESSO test.


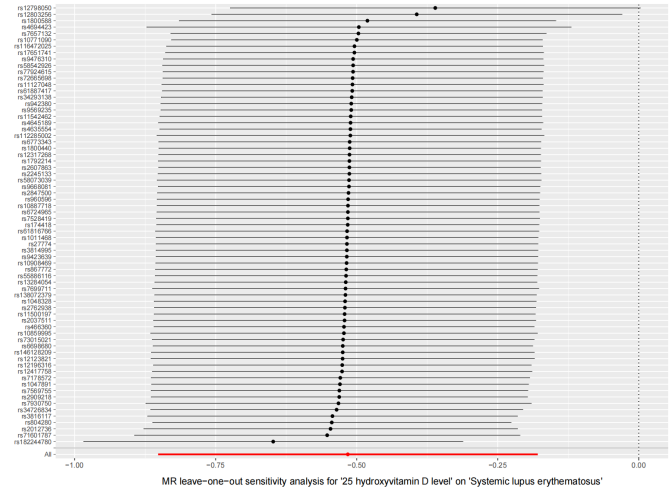


C


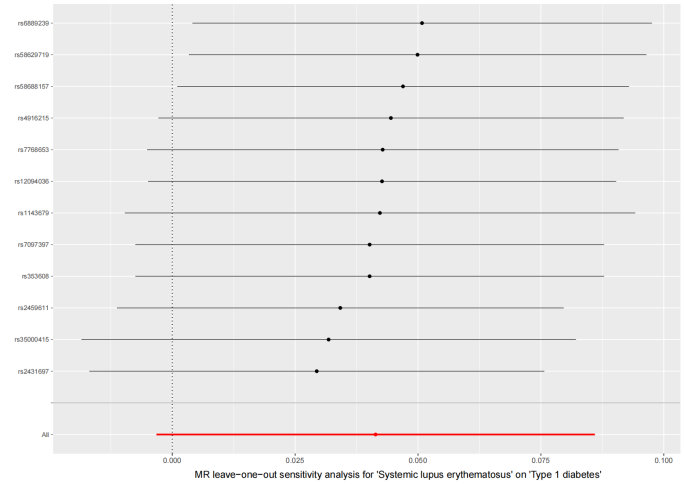


B


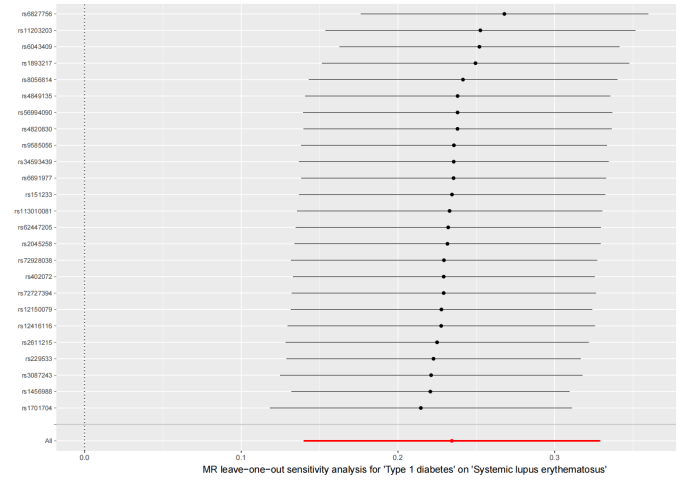


A


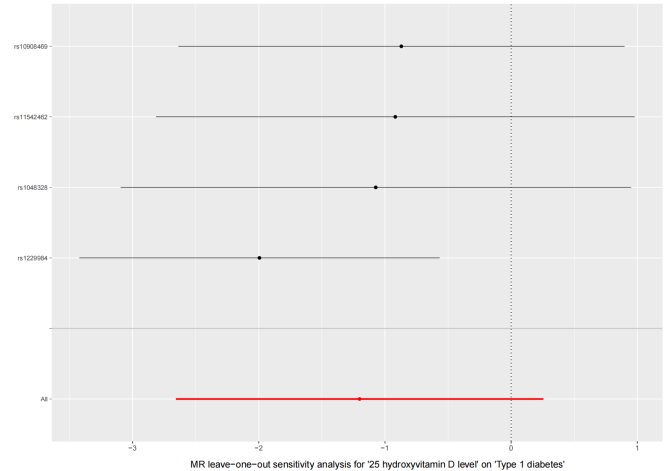


E


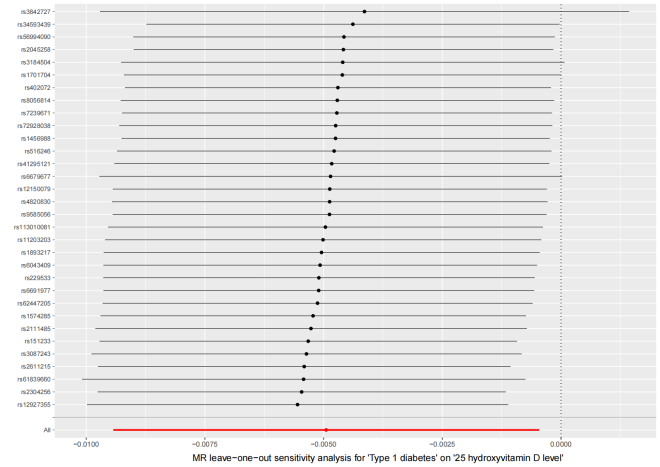


D

**S8 Fig. Leave-one-out sensitivity analysis for BIMR without outliers, a forest plot of causal estimates from the MR-IVW approach omitting each genetic variant in turn.** (A) Leave-one-out sensitivity analysis of the MR analysis of T1DM-associated SNPs on SLE without outliers. (B) Leave-one-out sensitivity analysis of the MR analysis of SLE-associated SNPs on T1DM without outliers. (C) Leave-one-out sensitivity analysis of the MR analysis of 25-OHD level-associated SNPs on SLE without outlier. (D) Leave-one-out sensitivity analysis of the MR analysis of T1DM-associated SNPs on 25-OHD level without outlier. (E) Leave-one-out sensitivity analysis of the MR analysis of 25-OHD level-associated SNPs on T1DM without outliers. BIMR, bidirectional mendelian randomization; SLE, systemic lupus erythematosus; T1DM, type 1 diabetes; 25-OHD, 25 hydroxyvitamin D.

*In MR analysis with SLE as exposure and 25-OHD level as result, there were no significant outliers identified by the MR-PRESSO test.


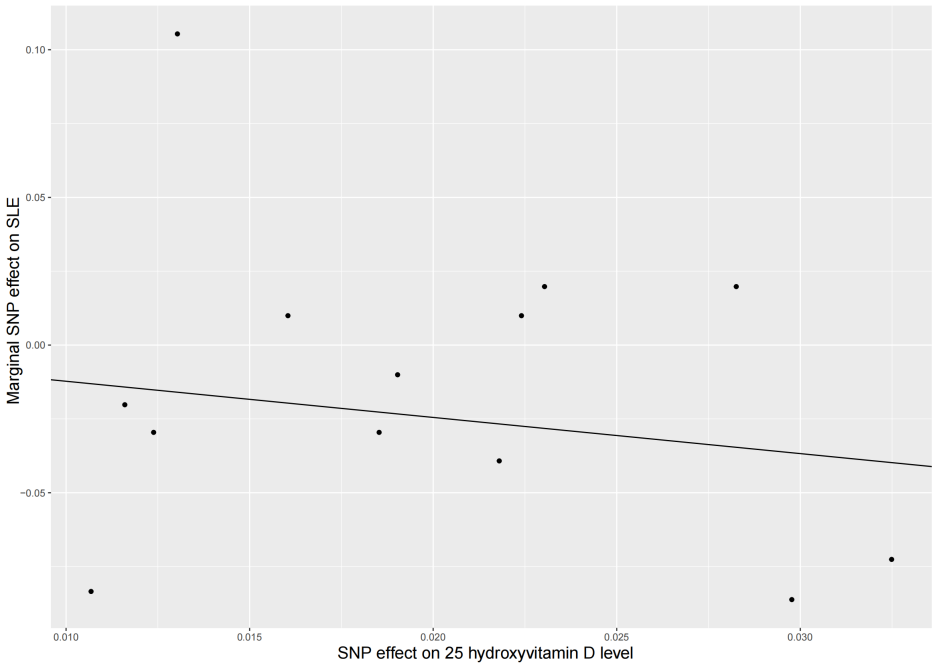


B


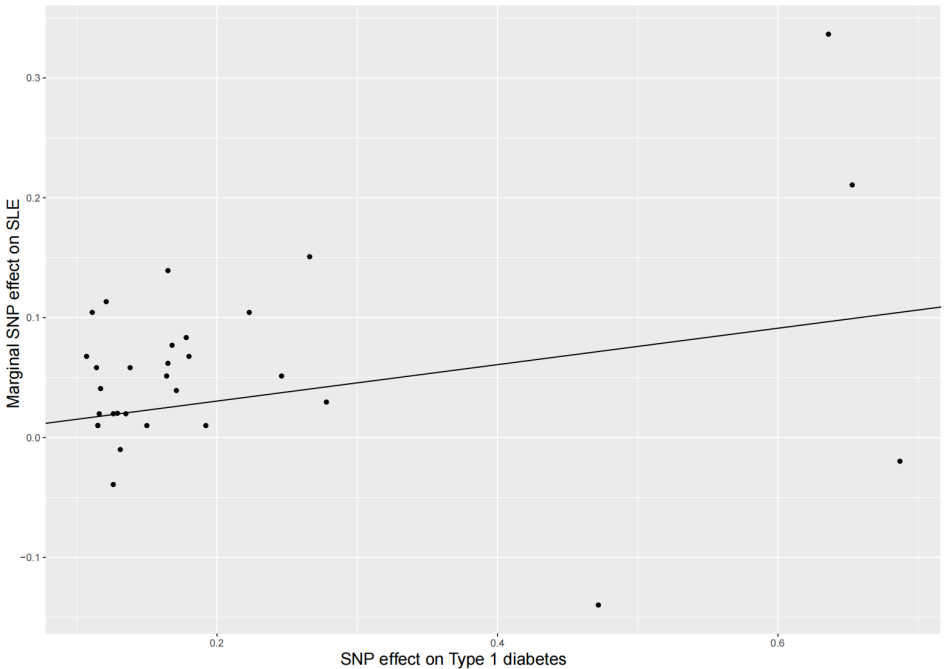


A


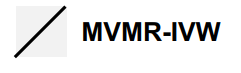


**S9 Fig. Scatter plot of SNP potential effects in MVMR analysis, with the slope of black line corresponding to estimated MR effect by MVMR-IVW.** The X-axis represents the effect size of SNPs on exposure respectively; the Y-axis represents the marginal effect size of SNPs on SLE. (A) Scatter plot of the MVMR analysis of T1DM-strongly associated SNPs on SLE. (B) Scatter plot of the MVMR analysis of 25VHD level-strongly associated SNPs on SLE. MVMR, multivariable mendelian randomization; SLE, systemic lupus erythematosus; T1DM, type 1 diabetes; 25-OHD, 25 hydroxyvitamin D.


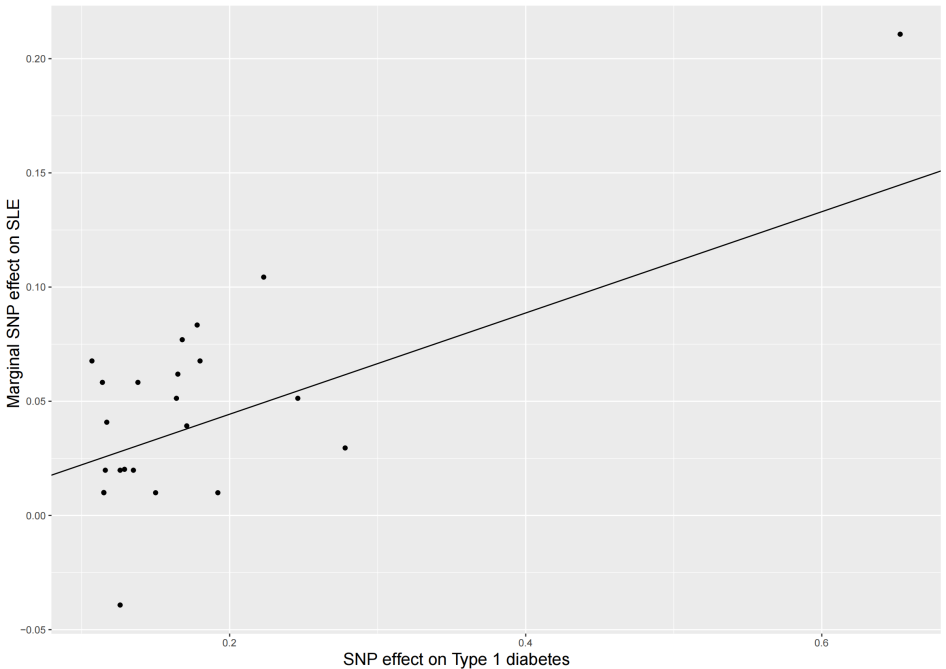


A


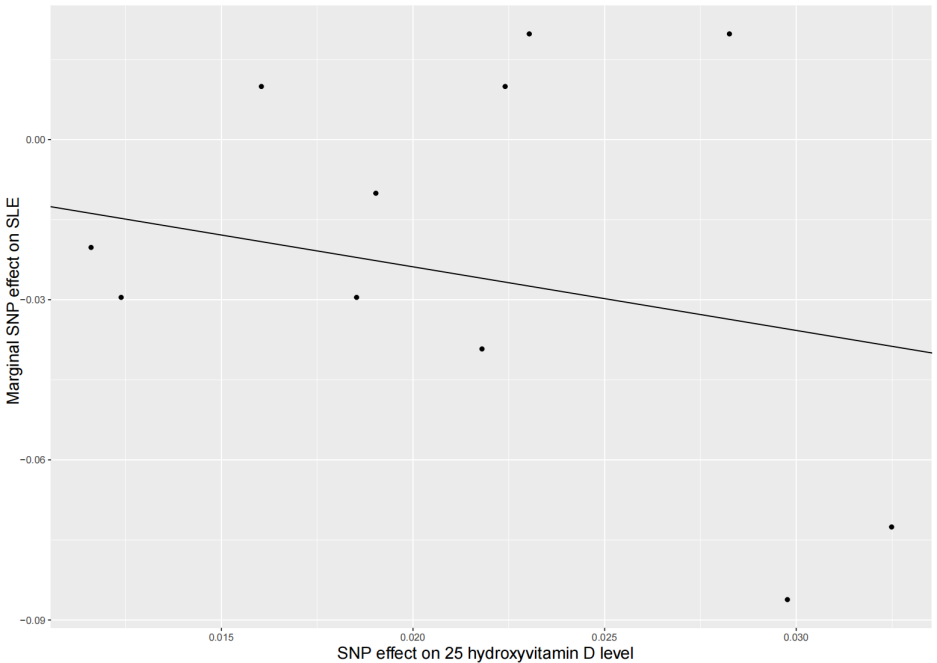


B


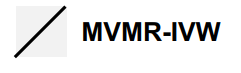


**S10 Fig. Scatter plot of SNP potential effects in MVMR-Lasso analysis, with the slope of black line corresponding to estimated MR effect by MVMR-IVW.** The X-axis represents the effect size of valid SNPs on exposure respectively; the Y-axis represents the marginal effect size of valid SNPs on SLE. (A) Scatter plot of the MVMR analysis of T1DM-strongly associated valid SNPs on SLE. (B) Scatter plot of the MVMR analysis of 25VHD-strongly associated valid SNPs on SLE. MVMR, multivariable mendelian randomization; SLE, systemic lupus erythematosus; T1DM, type 1 diabetes; 25-OHD, 25 hydroxyvitamin D.
