# Supplementary material for: A network causal relationship between type-1 diabetes mellitus, 25-hydroxyvitamin D level and systemic lupus erythematosus: Mendelian randomization study
Source: PLoS One. 2023 May 17;18(5):e0285915. doi: 10.1371/journal.pone.0285915 (PMC10191345; doi:10.1371/journal.pone.0285915)
Supplement: S2 File — (ZIP) [file pone.0285915.s002.zip › S2 File.docx]

| **S1 Table . Detailed information of instrumental variables in UVMR and BIMR** | | | | | | | | | | | | | |
| --- | --- | --- | --- | --- | --- | --- | --- | --- | --- | --- | --- | --- | --- |
| *Instrumental variabls of T1DM* | | | | | | | | | | | | | |
| Outcome | SNPid | Chr | Position | Effect Allele | Other Allele | Beta | Se | *P* | Sample Size | EAF | R2 | F-Stat | Gene |
| SLE | rs11203203 | 21 | 43836186 | A | G | 0.150 | 0.019 | 2.91×10^-15^ | 29652 | 0.375 | 5.53×10^-4^ | 29 | *CLEC16A* |
| SLE | rs113010081 | 3 | 46457412 | C | T | -0.164 | 0.030 | 4.59×10^-8^ | 29652 | 0.111 | 1.11×10^-4^ | 16 | *HORMAD2* |
| SLE | rs12150079 | 17 | 38025417 | A | G | 0.114 | 0.020 | 1.20×10^-8^ | 29652 | 0.319 | 2.67×10^-4^ | 14 | *UBASH3A* |
| SLE | rs12416116 | 10 | 90035654 | A | C | -0.165 | 0.021 | 3.93×10^-15^ | 29652 | 0.231 | 4.15×10^-4^ | 22 | *ZPBP2* |
| SLE | rs12927355 | 16 | 11194771 | T | C | -0.194 | 0.020 | 3.02×10^-22^ | 29652 | 0.718 | 7.20×10^-4^ | 38 | *IKZF4* |
| SLE | rs1456988 | 14 | 98488007 | T | G | -0.111 | 0.020 | 2.86×10^-8^ | 29652 | 0.285 | 2.37×10^-4^ | 13 | *ACOXL* |
| SLE | rs151233 | 16 | 28506428 | T | C | 0.171 | 0.026 | 4.80×10^-11^ | 29652 | 0.861 | 1.95×10^-4^ | 10 | *NCR* |
| SLE | rs1701704 | 12 | 56412487 | G | T | 0.223 | 0.019 | 8.25×10^-32^ | 29652 | 0.673 | 1.15×10^-3^ | 61 | *KIAA1109* |
| SLE | rs1893217 | 18 | 12809340 | G | A | 0.192 | 0.024 | 1.24×10^-15^ | 29652 | 0.843 | 3.21×10^-4^ | 17 | *NCR* |
| SLE | rs2045258 | 6 | 126674354 | G | A | 0.117 | 0.019 | 7.37×10^-10^ | 29652 | 0.532 | 3.57×10^-4^ | 19 | *CAMSAP2* |
| SLE | rs2111485* | 2 | 163110536 | G | A | 0.165 | 0.019 | 3.81×10^-18^ | 29652 | 0.397 | 6.83×10^-4^ | 36 | *CTLA4* |
| SLE | rs229533 | 22 | 37587111 | C | A | 0.107 | 0.019 | 1.79×10^-8^ | 29652 | 0.574 | 2.93×10^-4^ | 16 | *TYK2* |
| SLE | rs2304256* | 19 | 10475652 | A | C | -0.139 | 0.021 | 3.61×10^-11^ | 29652 | 0.716 | 3.37×10^-4^ | 18 | *PTPN2* |
| SLE | rs2611215 | 4 | 166574267 | G | A | -0.168 | 0.025 | 1.82×10^-11^ | 29652 | 0.163 | 2.33×10^-4^ | 12 | *APOBR* |
| SLE | rs3087243 | 2 | 204738919 | A | G | -0.178 | 0.019 | 7.36×10^-21^ | 29652 | 0.546 | 8.23×10^-4^ | 44 | *IKZF1* |
| SLE | rs3184504* | 12 | 111884608 | C | T | -0.266 | 0.019 | 1.56×10^-44^ | 29652 | 0.501 | 1.85×10^-3^ | 98 | *CTSH* |
| SLE | rs34593439 | 15 | 79234957 | A | G | -0.246 | 0.033 | 9.02×10^-14^ | 29652 | 0.893 | 2.01×10^-4^ | 11 | *CTRB1* |
| SLE | rs3842727* | 11 | 2184848 | T | G | 0.687 | 0.023 | 4.89×10^-196^ | 29652 | 0.311 | 7.23×10^-4^ | 385 | *NCR* |
| SLE | rs402072 | 19 | 47219122 | C | T | -0.142 | 0.026 | 4.72×10^-8^ | 29652 | 0.841 | 1.51×10^-4^ | 10 | *NCR* |
| SLE | rs4820830 | 22 | 30531091 | T | C | -0.135 | 0.019 | 1.20×10^-12^ | 29652 | 0.347 | 4.33×10^-4^ | 23 | *BACH2* |
| SLE | rs4849135 | 2 | 111615079 | G | T | 0.115 | 0.021 | 4.35×10^-8^ | 29652 | 0.281 | 2.29×10^-4^ | 12 | *RNLS* |
| SLE | rs516246* | 19 | 49206172 | T | C | 0.143 | 0.019 | 5.22×10^-14^ | 29652 | 0.532 | 5.34×10^-4^ | 28 | *PHTF1* |
| SLE | rs56994090 | 14 | 101306447 | C | T | -0.129 | 0.019 | 1.13×10^-11^ | 29652 | 0.579 | 4.25×10^-4^ | 22 | *TH* |
| SLE | rs6043409 | 20 | 1616206 | G | A | 0.126 | 0.020 | 2.98×10^-10^ | 29652 | 0.335 | 3.34×10^-4^ | 18 | *RASGRP1* |
| SLE | rs61839660* | 10 | 6094697 | T | C | -0.472 | 0.036 | 2.84×10^-39^ | 29652 | 0.91 | 5.35×10^-4^ | 28 | *IL2RA* |
| SLE | rs62447205 | 7 | 50465830 | G | A | -0.117 | 0.021 | 2.53×10^-8^ | 29652 | 0.726 | 2.33×10^-4^ | 12 | *SIRPG* |
| SLE | rs6679677* | 1 | 114303808 | A | C | 0.636 | 0.027 | 1.10×10^-122^ | 29652 | 0.904 | 1.82×10^-4^ | 96 | *C1QTNF6* |
| SLE | rs6691977 | 1 | 200814959 | C | T | 0.126 | 0.023 | 4.30×10^-8^ | 29652 | 0.777 | 1.97×10^-4^ | 10 | *LOC107984558* |
| SLE | rs6827756 | 4 | 123184411 | C | T | -0.131 | 0.019 | 5.40×10^-12^ | 29652 | 0.382 | 4.24×10^-4^ | 22 | *FUT2* |
| SLE | rs7239671* | 18 | 67523260 | G | A | 0.121 | 0.018 | 1.79×10^-11^ | 29652 | 0.537 | 4.25×10^-4^ | 22 | *LOC105377068* |
| SLE | rs72727394 | 15 | 38847022 | T | C | 0.138 | 0.022 | 3.55×10^-10^ | 29652 | 0.796 | 2.42×10^-4^ | 13 | *PRKD2* |
| SLE | rs72928038 | 6 | 90976768 | A | G | 0.180 | 0.024 | 6.38×10^-14^ | 29652 | 0.849 | 2.73×10^-4^ | 14 | *MEG3* |
| SLE | rs8056814 | 16 | 75252327 | A | G | 0.278 | 0.031 | 3.03×10^-19^ | 29652 | 0.915 | 2.36×10^-4^ | 12 | *SH2B* |
| SLE | rs9585056 | 13 | 100081766 | T | C | -0.116 | 0.021 | 3.32×10^-8^ | 29652 | 0.244 | 2.13×10^-4^ | 11 | *CENPW* |
| 25-OHD level | rs11203203 | 21 | 43836186 | A | G | 0.150 | 0.019 | 2.91×10^-15^ | 29652 | 0.625 | 6.20×10^-5^ | 29 | *UBASH3A* |
| 25-OHD level | rs113010081 | 3 | 46457412 | C | T | -0.164 | 0.030 | 4.59×10^-8^ | 29652 | 0.889 | 1.25×10^-5^ | 16 | *NCR* |
| 25-OHD level | rs12150079 | 17 | 38025417 | A | G | 0.114 | 0.020 | 1.20×10^-8^ | 29652 | 0.681 | 3.00×10^-5^ | 14 | *ZPBP2* |
| 25-OHD level | rs12927355 | 16 | 11194771 | T | C | -0.194 | 0.020 | 3.02×10^-22^ | 29652 | 0.718 | 8.09×10^-5^ | 38 | *CLEC16A* |
| 25-OHD level | rs1456988 | 14 | 98488007 | T | G | -0.111 | 0.020 | 2.86×10^-8^ | 29652 | 0.285 | 2.66×10^-5^ | 13 | *NCR* |
| 25-OHD level | rs151233 | 16 | 28506428 | T | C | 0.171 | 0.026 | 4.80×10^-11^ | 29652 | 0.861 | 2.19×10^-5^ | 10 | *APOBR* |
| 25-OHD level | rs1574285 | 9 | 4283137 | T | G | -0.115 | 0.019 | 1.42×10^-9^ | 29652 | 0.425 | 3.80×10^-5^ | 18 | *GLIS3* |
| 25-OHD level | rs1701704 | 12 | 56412487 | G | T | 0.223 | 0.019 | 8.25×10^-32^ | 29652 | 0.673 | 1.29×10^-4^ | 61 | *IKZF4* |
| 25-OHD level | rs1893217 | 18 | 12809340 | G | A | 0.192 | 0.024 | 1.24×10^-15^ | 29652 | 0.843 | 3.60×10^-5^ | 17 | *PTPN2* |
| 25-OHD level | rs2045258 | 6 | 126674354 | G | A | 0.117 | 0.019 | 7.37×10^-10^ | 29652 | 0.532 | 4.01×10^-5^ | 19 | *CENPW* |
| 25-OHD level | rs2111485 | 2 | 163110536 | G | A | 0.165 | 0.019 | 3.81×10^-18^ | 29652 | 0.397 | 7.67×10^-5^ | 36 | *NCR* |
| 25-OHD level | rs229533 | 22 | 37587111 | C | A | 0.107 | 0.019 | 1.79×10^-8^ | 29652 | 0.574 | 3.29×10^-5^ | 16 | *C1QTNF6* |
| 25-OHD level | rs2304256 | 19 | 10475652 | A | C | -0.139 | 0.021 | 3.61×10^-11^ | 29652 | 0.716 | 3.78×10^-5^ | 18 | *TYK2* |
| 25-OHD level | rs2611215 | 4 | 166574267 | G | A | -0.168 | 0.025 | 1.82×10^-11^ | 29652 | 0.163 | 2.61×10^-5^ | 12 | *NCR* |
| 25-OHD level | rs3087243 | 2 | 204738919 | A | G | -0.178 | 0.019 | 7.36×10^-21^ | 29652 | 0.546 | 9.24×10^-5^ | 44 | *CTLA4* |
| 25-OHD level | rs3184504 | 12 | 111884608 | C | T | -0.266 | 0.019 | 1.56×10^-44^ | 29652 | 0.501 | 2.08×10^-4^ | 98 | *SH2B3* |
| 25-OHD level | rs34593439 | 15 | 79234957 | A | G | -0.246 | 0.033 | 9.02×10^-14^ | 29652 | 0.893 | 2.25×10^-5^ | 11 | *CTSH* |
| 25-OHD level | rs3842727 | 11 | 2184848 | T | G | 0.687 | 0.023 | 4.89×10^-196^ | 29652 | 0.311 | 8.12×10^-4^ | 383 | *TH* |
| 25-OHD level | rs402072 | 19 | 47219122 | C | T | -0.142 | 0.026 | 4.72×10^-8^ | 29652 | 0.86 | 1.53×10^-5^ | 10 | *PRKD2* |
| 25-OHD level | rs41295121 | 10 | 6129643 | T | C | -0.653 | 0.112 | 5.53×10^-9^ | 29652 | 0.991 | 1.28×10^-6^ | 11 | *RBM17* |
| 25-OHD level | rs4820830 | 22 | 30531091 | T | C | -0.135 | 0.019 | 1.20×10^-12^ | 29652 | 0.347 | 4.86×10^-5^ | 23 | *HORMAD2* |
| 25-OHD level | rs516246 | 19 | 49206172 | T | C | 0.143 | 0.019 | 5.22×10^-14^ | 29652 | 0.532 | 5.99×10^-5^ | 28 | *FUT2* |
| 25-OHD level | rs56994090 | 14 | 101306447 | C | T | -0.129 | 0.019 | 1.13×10^-11^ | 29652 | 0.579 | 4.77×10^-5^ | 22 | *MEG3* |
| 25-OHD level | rs6043409 | 20 | 1616206 | G | A | 0.126 | 0.020 | 2.98×10^-10^ | 29652 | 0.335 | 3.75×10^-5^ | 18 | *SIRPG* |
| 25-OHD level | rs61839660 | 10 | 6094697 | T | C | -0.472 | 0.036 | 2.84×10^-39^ | 29652 | 0.91 | 6.01×10^-5^ | 28 | *IL2RA* |
| 25-OHD level | rs62447205 | 7 | 50465830 | G | A | -0.117 | 0.021 | 2.53×10^-8^ | 29652 | 0.726 | 2.62×10^-5^ | 12 | *IKZF1* |
| 25-OHD level | rs6679677 | 1 | 114303808 | A | C | 0.636 | 0.027 | 1.10×10^-122^ | 29652 | 0.904 | 2.05×10^-5^ | 96 | *PHTF1* |
| 25-OHD level | rs6691977 | 1 | 200814959 | C | T | 0.126 | 0.023 | 4.30×10^-8^ | 29652 | 0.777 | 2.21×10^-5^ | 10 | *CAMSAP2* |
| 25-OHD level | rs7239671 | 18 | 67523260 | G | A | 0.121 | 0.018 | 1.79×10^-11^ | 29652 | 0.537 | 4.77×10^-5^ | 22 | *CD226* |
| 25-OHD level | rs72727394* | 15 | 38847022 | T | C | 0.138 | 0.022 | 3.55×10^-10^ | 29652 | 0.796 | 2.72×10^-5^ | 13 | *RASGRP1* |
| 25-OHD level | rs72928038 | 6 | 90976768 | A | G | 0.180 | 0.024 | 6.38×10^-14^ | 29652 | 0.849 | 3.07×10^-5^ | 14 | *BACH2* |
| 25-OHD level | rs8056814 | 16 | 75252327 | A | G | 0.278 | 0.031 | 3.03×10^-19^ | 29652 | 0.915 | 2.65×10^-5^ | 12 | *CTRB1* |
| 25-OHD level | rs9585056 | 13 | 100081766 | T | C | -0.116 | 0.021 | 3.32×10^-8^ | 29652 | 0.244 | 2.39×10^-5^ | 11 | *LOC107984558* |
| *Instrumental variabls of SLE* | | | | | | | | | | | | | |
| T1DM | rs1143679 | 16 | 31276811 | A | G | 0.582 | 0.040 | 5.03×10^-48^ | 23210 | 0.878 | 8.59×10^-4^ | 45 | *ITGAM* |
| T1DM | rs12094036 | 1 | 183558174 | C | T | -0.329 | 0.058 | 1.37×10^-8^ | 23210 | 0.926 | 8.38×10^-5^ | 14 | *NCF2* |
| T1DM | rs2431697 | 5 | 159879978 | C | T | -0.223 | 0.029 | 2.60×10^-14^ | 23210 | 0.564 | 5.40×10^-4^ | 29 | *NCR* |
| T1DM | rs2459611 | 2 | 191939187 | T | C | 0.261 | 0.045 | 7.62×10^-9^ | 23210 | 0.113 | 1.26×10^-4^ | 10 | *STAT4* |
| T1DM | rs35000415 | 7 | 128585616 | T | C | 0.588 | 0.042 | 1.86×10^-45^ | 23210 | 0.873 | 8.39×10^-4^ | 44 | *IRF5* |
| T1DM | rs353608 | 11 | 35101738 | G | A | 0.186 | 0.028 | 2.93×10^-11^ | 23210 | 0.478 | 4.17×10^-4^ | 22 | *NCR* |
| T1DM | rs4274624* | 2 | 191958656 | T | C | -0.560 | 0.033 | 9.73×10^-66^ | 23210 | 0.222 | 1.92×10^-3^ | 101 | *STAT4* |
| T1DM | rs4916215 | 1 | 173314540 | T | C | 0.223 | 0.034 | 5.07×10^-11^ | 23210 | 0.26 | 3.14×10^-4^ | 17 | *TNFSF4* |
| T1DM | rs58688157 | 11 | 625085 | G | A | -0.223 | 0.034 | 2.97×10^-11^ | 23210 | 0.725 | 3.34×10^-4^ | 18 | *CDHR5* |
| T1DM | rs58721818* | 6 | 138243739 | T | C | 0.658 | 0.076 | 3.38×10^-18^ | 23210 | 0.976 | 6.66×10^-5^ | 14 | *NCR* |
| T1DM | rs58629719 | 5 | 150457771 | C | T | 0.278 | 0.032 | 2.19×10^-18^ | 23210 | 0.75 | 5.43×10^-4^ | 29 | *TNIP1* |
| T1DM | rs6889239 | 5 | 150457771 | C | T | 0.278 | 0.032 | 2.19×10^-18^ | 23210 | 0.623 | 6.80×10^-4^ | 36 | *TNIP1* |
| T1DM | rs7097397 | 10 | 50025396 | A | G | -0.186 | 0.029 | 8.60×10^-11^ | 23210 | 0.62 | 3.75×10^-4^ | 20 | *WDFY4* |
| T1DM | rs7768653 | 6 | 106574794 | T | C | -0.207 | 0.030 | 3.11×10^-12^ | 23210 | 0.394 | 4.39×10^-4^ | 23 | *NCR* |
| 25-OHD level | rs10048743 | 2 | 213890232 | T | G | -0.231 | 0.041 | 2.04×10^-8^ | 23210 | 0.14 | 3.26×10^-4^ | 18 | *IKZF2* |
| 25-OHD level | rs10200680 | 2 | 223961877 | T | C | -0.248 | 0.042 | 4.96×10^-9^ | 23210 | 0.874 | 3.25×10^-4^ | 10 | *NCR* |
| 25-OHD level | rs1078324 | 5 | 149202268 | A | C | -0.713 | 0.078 | 7.11×10^-20^ | 23210 | 0.965 | 2.43×10^-4^ | 11 | *PPARGC1B* |
| 25-OHD level | rs10912578 | 1 | 173251856 | G | A | -0.247 | 0.031 | 1.65×10^-15^ | 23210 | 0.315 | 1.18×10^-3^ | 27 | *TNFSF4* |
| 25-OHD level | rs1143679 | 16 | 31276811 | A | G | 0.582 | 0.040 | 5.03×10^-48^ | 23210 | 0.878 | 1.96×10^-3^ | 46 | *ITGAM* |
| 25-OHD level | rs12094036 | 1 | 183558174 | C | T | -0.329 | 0.058 | 1.37×10^-8^ | 23210 | 0.926 | 1.91×10^-4^ | 14 | *NCF2* |
| 25-OHD level | rs12524498 | 6 | 31444187 | T | G | -0.673 | 0.121 | 2.48×10^-8^ | 23210 | 0.981 | 5.05×10^-5^ | 11 | *NCR* |
| 25-OHD level | rs13019891 | 2 | 113829869 | T | G | -0.562 | 0.029 | 1.65×10^-83^ | 23210 | 0.549 | 8.00×10^-3^ | 187 | *IL1F10* |
| 25-OHD level | rs13136219 | 4 | 102743687 | T | C | -0.174 | 0.028 | 3.50×10^-10^ | 23210 | 0.635 | 7.87×10^-4^ | 18 | *BANK1* |
| 25-OHD level | rs13332649 | 16 | 85966683 | G | A | -0.315 | 0.038 | 5.43×10^-17^ | 23210 | 0.778 | 1.04×10^-3^ | 24 | *NCR* |
| 25-OHD level | rs1464446 | 3 | 146601295 | T | G | -0.329 | 0.040 | 2.79×10^-16^ | 23210 | 0.82 | 8.52×10^-4^ | 20 | *NCR* |
| 25-OHD level | rs17849501 | 1 | 183542323 | T | C | 0.811 | 0.050 | 1.81×10^-59^ | 23210 | 0.948 | 1.12×10^-3^ | 26 | *NCF2* |
| 25-OHD level | rs2459611 | 2 | 191939187 | T | C | 0.261 | 0.045 | 7.62×10^-9^ | 23210 | 0.113 | 2.88×10^-4^ | 17 | *STAT4* |
| 25-OHD level | rs268124 | 2 | 65654364 | T | C | 0.186 | 0.032 | 8.60×10^-9^ | 23210 | 0.334 | 6.35×10^-4^ | 15 | *SPRED2* |
| 25-OHD level | rs28361029 | 6 | 31220203 | A | G | -0.386 | 0.061 | 3.27×10^-10^ | 23210 | 0.975 | 8.22×10^-5^ | 12 | *NCR* |
| 25-OHD level | rs34703115 | 2 | 40282854 | C | T | -0.616 | 0.105 | 4.08×10^-9^ | 23210 | 0.96 | 1.15×10^-4^ | 13 | *SLC8A1-AS1* |
| 25-OHD level | rs35000415 | 7 | 128585616 | T | C | 0.588 | 0.042 | 1.86×10^-45^ | 23210 | 0.873 | 1.91×10^-3^ | 44 | *IRF5* |
| 25-OHD level | rs35251378 | 19 | 10459969 | A | G | -0.236 | 0.032 | 3.61×10^-13^ | 23210 | 0.716 | 9.26×10^-4^ | 22 | *NCR* |
| 25-OHD level | rs353608 | 11 | 35101738 | G | A | 0.186 | 0.028 | 2.93×10^-11^ | 23210 | 0.478 | 9.51×10^-4^ | 22 | *NCR* |
| 25-OHD level | rs389884 | 6 | 31940897 | G | A | 0.928 | 0.043 | 2.92×10^-`102^ | 23210 | 0.895 | 3.75×10^-3^ | 87 | *DXO* |
| 25-OHD level | rs4274624 | 2 | 191958656 | T | C | -0.560 | 0.033 | 9.73×10^-66^ | 23210 | 0.222 | 4.36×10^-3^ | 102 | *STAT4* |
| 25-OHD level | rs4388254 | 5 | 133428601 | T | C | 0.378 | 0.060 | 3.71×10^-10^ | 23210 | 0.956 | 1.43×10^-4^ | 13 | *LOC105379185* |
| 25-OHD level | rs4661543 | 1 | 15229101 | G | T | 0.274 | 0.042 | 9.40×10^-11^ | 23210 | 0.132 | 4.14×10^-4^ | 10 | *KAZN* |
| 25-OHD level | rs4916215 | 1 | 173314540 | T | C | 0.223 | 0.034 | 5.07×10^-11^ | 23210 | 0.26 | 7.15×10^-4^ | 17 | *TNFSF4* |
| 25-OHD level | rs58688157 | 11 | 625085 | G | A | -0.223 | 0.034 | 2.97×10^-11^ | 23210 | 0.725 | 7.60×10^-4^ | 18 | *CDHR5* |
| 25-OHD level | rs58721818 | 6 | 138243739 | T | C | 0.658 | 0.076 | 3.38×10^-18^ | 23210 | 0.976 | 1.52×10^-4^ | 14 | *NCR* |
| 25-OHD level | rs597808 | 12 | 111973358 | G | A | -0.163 | 0.029 | 3.51×10^-8^ | 23210 | 0.474 | 6.53×10^-4^ | 15 | *ATXN2* |
| 25-OHD level | rs6671847 | 1 | 161478810 | A | G | 0.199 | 0.029 | 6.64×10^-12^ | 23210 | 0.536 | 1.01×10^-3^ | 23 | *FCGR2A* |
| 25-OHD level | rs6679677 | 1 | 114303808 | A | C | 0.336 | 0.046 | 4.55×10^-13^ | 23210 | 0.904 | 3.92×10^-4^ | 19 | *PHTF1* |
| 25-OHD level | rs6889239 | 5 | 150457771 | C | T | 0.278 | 0.032 | 2.19×10^-18^ | 23210 | 0.75 | 1.24×10^-3^ | 29 | *TNIP1* |
| 25-OHD level | rs7097397 | 10 | 50025396 | A | G | -0.186 | 0.029 | 8.60×10^-11^ | 23210 | 0.62 | 8.55×10^-4^ | 20 | *WDFY4* |
| 25-OHD level | rs73050535 | 12 | 5012503 | T | C | -0.713 | 0.124 | 9.11×10^-9^ | 23210 | 0.992 | 2.14×10^-5^ | 10 | *NCR* |
| 25-OHD level | rs73068668 | 19 | 55763262 | A | G | -0.315 | 0.057 | 4.40×10^-8^ | 23210 | 0.957 | 1.06×10^-4^ | 12 | *PPP6R1* |
| 25-OHD level | rs7768653 | 6 | 106574794 | T | C | -0.207 | 0.030 | 3.11×10^-12^ | 23210 | 0.394 | 1.00×10^-3^ | 23 | *NCR* |
| 25-OHD level | rs7823055 | 8 | 55511676 | T | G | -0.351 | 0.029 | 1.64×10^-34^ | 23210 | 0.423 | 4.84×10^-4^ | 11 | *RP1* |
| 25-OHD level | rs7899626 | 10 | 63825561 | T | C | 0.182 | 0.033 | 4.19×10^-8^ | 23210 | 0.686 | 5.58×10^-4^ | 13 | *ARID5B* |
| 25-OHD level | rs9274357 | 6 | 32632457 | T | C | 0.457 | 0.035 | 1.28×10^-38^ | 23210 | 0.776 | 2.53×10^-3^ | 59 | *HLA-DQB1* |
| 25-OHD level | rs9852014 | 3 | 129084581 | G | A | 0.621 | 0.049 | 2.26×10^-36^ | 23210 | 0.923 | 9.72×10^-4^ | 23 | *NCR* |
| *Instrumental variabls of 25-OHD level* | | | | | | | | | | | | | |
| SLE | rs1011468 | 7 | 104613791 | A | G | -0.014 | 0.002 | 1.35×10^-12^ | 441,291 | 0.476 | 5.40×10^-5^ | 25 | *KMT2E* |
| SLE | rs1047891 | 2 | 211540507 | A | C | -0.014 | 0.002 | 1.16×10^-11^ | 441,291 | 0.316 | 4.29×10^-5^ | 20 | *CPS1* |
| SLE | rs1048328 | 19 | 51527364 | A | G | 0.028 | 0.004 | 4.12×10^-15^ | 441,291 | 0.08 | 1.95×10^-5^ | 19 | *KLK11* |
| SLE | rs10771090 | 12 | 24588749 | G | A | -0.011 | 0.002 | 4.32×10^-8^ | 441,291 | 0.475 | 3.22×10^-5^ | 15 | *SOX5* |
| SLE | rs10859995 | 12 | 96375682 | C | T | -0.039 | 0.002 | 7.03×10^-89^ | 441,291 | 0.581 | 4.19×10^-4^ | 195 | *HAL* |
| SLE | rs10887718 | 10 | 82042624 | T | C | -0.012 | 0.002 | 1.44×10^-10^ | 441,291 | 0.527 | 4.41×10^-5^ | 20 | *MAT1A* |
| SLE | rs10908469 | 1 | 155468732 | C | A | 0.016 | 0.002 | 2.20×10^-13^ | 441,291 | 0.27 | 4.57×10^-5^ | 21 | *ASH1L* |
| SLE | rs11127048 | 2 | 27752463 | A | G | 0.018 | 0.002 | 6.41×10^-19^ | 441,291 | 0.617 | 8.04×10^-5^ | 37 | *NCR* |
| SLE | rs112285002 | 19 | 48374320 | T | C | 0.060 | 0.003 | 1.77×10^-110^ | 441,291 | 0.16 | 2.88×10^-4^ | 134 | *SULT2A1* |
| SLE | rs11500197 | 11 | 27707409 | A | G | -0.012 | 0.002 | 3.99×10^-8^ | 441,291 | 0.262 | 2.51×10^-5^ | 12 | *BDNF-AS* |
| SLE | rs11542462 | 16 | 82033810 | A | G | -0.022 | 0.003 | 3.39×10^-14^ | 441,291 | 0.134 | 2.87×10^-5^ | 13 | *SDR42E1* |
| SLE | rs116472025 | 4 | 99839056 | A | G | 0.037 | 0.006 | 6.83×10^-10^ | 441,291 | 0.028 | 4.44×10^-6^ | 12 | *EIF4E* |
| SLE | rs12123821 | 1 | 152179152 | T | C | 0.074 | 0.005 | 2.25×10^-59^ | 441,291 | 0.048 | 5.19×10^-5^ | 24 | *LOC112268240* |
| SLE | rs12196316 | 6 | 22757900 | C | T | -0.012 | 0.002 | 5.51×10^-9^ | 441,291 | 0.301 | 3.08×10^-5^ | 14 | *NCR* |
| SLE | rs12317268 | 12 | 21352541 | G | A | -0.019 | 0.003 | 9.15×10^-12^ | 441,291 | 0.152 | 2.58×10^-5^ | 12 | *SLCO1B1* |
| SLE | rs12417758 | 11 | 66076360 | C | T | 0.012 | 0.002 | 2.08×10^-9^ | 441,291 | 0.456 | 3.83×10^-5^ | 18 | *NCR* |
| SLE | rs12798050 | 11 | 71223256 | T | C | 0.107 | 0.003 | 1.00×10^-200^ | 441,291 | 0.824 | 1.06×10^-3^ | 493 | *NCR* |
| SLE | rs12803256 | 11 | 71132868 | G | A | 0.100 | 0.002 | 1.00×10^-200^ | 441,291 | 0.771 | 1.42×10^-3^ | 659 | *ACTE1P* |
| SLE | rs13284054 | 9 | 107669073 | C | T | 0.017 | 0.003 | 8.39×10^-9^ | 441,291 | 0.124 | 1.56×10^-5^ | 17 | *ABCA1* |
| SLE | rs138072379 | 11 | 14332994 | T | C | 0.043 | 0.007 | 6.56×10^-10^ | 441,291 | 0.021 | 3.35×10^-6^ | 12 | *RRAS2* |
| SLE | rs146128209 | 11 | 14683683 | G | A | -0.047 | 0.004 | 1.03×10^-35^ | 441,291 | 0.071 | 4.44×10^-5^ | 21 | *PDE3B* |
| SLE | rs174418 | 15 | 58687603 | C | T | 0.022 | 0.002 | 2.99×10^-28^ | 441,291 | 0.595 | 1.26×10^-4^ | 59 | *NCR* |
| SLE | rs17651741 | 15 | 38869666 | A | G | -0.013 | 0.002 | 4.90×10^-8^ | 441,291 | 0.194 | 2.00×10^-5^ | 10 | *LOC107984739* |
| SLE | rs1792214 | 11 | 71069311 | G | T | 0.016 | 0.002 | 2.09×10^-15^ | 441,291 | 0.332 | 6.02×10^-5^ | 28 | *NCR* |
| SLE | rs1800440 | 2 | 38298139 | C | T | -0.014 | 0.002 | 2.06×10^-8^ | 441,291 | 0.186 | 2.05×10^-5^ | 10 | *CYP1B1* |
| SLE | rs1800588 | 15 | 58723675 | T | C | -0.030 | 0.002 | 2.65×10^-36^ | 441,291 | 0.215 | 1.15×10^-4^ | 53 | *LIPC* |
| SLE | rs182244780 | 11 | 14385531 | A | G | -0.335 | 0.009 | 1.00×10^-200^ | 441,291 | 0.013 | 8.88×10^-5^ | 41 | *RRAS2* |
| SLE | rs2012736 | 2 | 234622379 | A | C | -0.046 | 0.004 | 7.65×10^-37^ | 441,291 | 0.082 | 5.18×10^-5^ | 24 | *UGT1A10* |
| SLE | rs2037511 | 18 | 61366207 | A | G | 0.016 | 0.003 | 9.29×10^-10^ | 441,291 | 0.165 | 2.23×10^-5^ | 10 | *NCR* |
| SLE | rs2245133 | 6 | 131931092 | C | T | -0.021 | 0.003 | 1.62×10^-15^ | 441,291 | 0.166 | 3.79×10^-5^ | 18 | *MED23* |
| SLE | rs2607863 | 10 | 88024443 | C | T | -0.025 | 0.004 | 1.02×10^-8^ | 441,291 | 0.942 | 7.70×10^-5^ | 11 | *GRID1* |
| SLE | rs2762938 | 20 | 52778335 | A | G | 0.013 | 0.002 | 4.63×10^-11^ | 441,291 | 0.585 | 4.53×10^-5^ | 21 | *CYP24A1* |
| SLE | rs27774 | 5 | 87959033 | A | G | -0.012 | 0.002 | 1.64×10^-8^ | 441,291 | 0.292 | 2.84×10^-5^ | 13 | *LINC00461* |
| SLE | rs2847500 | 11 | 120114421 | A | G | -0.021 | 0.003 | 7.79×10^-13^ | 441,291 | 0.124 | 2.41×10^-5^ | 11 | *POU2F3* |
| SLE | rs2909218 | 17 | 66464546 | T | C | 0.017 | 0.002 | 2.81×10^-12^ | 441,291 | 0.793 | 3.45×10^-5^ | 16 | *PRKAR1A* |
| SLE | rs34293138 | 3 | 49579017 | C | T | -0.012 | 0.002 | 3.82×10^-9^ | 441,291 | 0.307 | 3.18×10^-5^ | 15 | *NCR* |
| SLE | rs34726834 | 8 | 25889606 | T | C | 0.014 | 0.002 | 6.65×10^-10^ | 441,291 | 0.254 | 3.11×10^-5^ | 14 | *EBF2* |
| SLE | rs3814995 | 19 | 36342212 | T | C | -0.015 | 0.002 | 2.83×10^-12^ | 441,291 | 0.312 | 4.51×10^-5^ | 21 | *NPHS1* |
| SLE | rs3816117 | 16 | 56996158 | C | T | -0.016 | 0.002 | 2.63×10^-17^ | 441,291 | 0.487 | 7.70×10^-5^ | 36 | *CETP* |
| SLE | rs4635554 | 2 | 21389659 | G | T | -0.013 | 0.002 | 6.44×10^-10^ | 441,291 | 0.336 | 3.67×10^-5^ | 17 | *NCR* |
| SLE | rs4645189 | 4 | 72359910 | T | C | 0.023 | 0.003 | 2.79×10^-19^ | 441,291 | 0.829 | 4.93×10^-5^ | 23 | *SLC4A4* |
| SLE | rs466360 | 5 | 143898025 | A | G | -0.011 | 0.002 | 1.13×10^-8^ | 441,291 | 0.392 | 3.35×10^-5^ | 16 | *NCR* |
| SLE | rs4694423 | 4 | 72554159 | A | C | -0.097 | 0.002 | 1.00×10^-200^ | 441,291 | 0.417 | 2.56×10^-3^ | 1193 | *NCR* |
| SLE | rs55886116 | 2 | 63068489 | T | G | 0.014 | 0.002 | 5.13×10^-9^ | 441,291 | 0.201 | 2.36×10^-5^ | 11 | *EHBP1* |
| SLE | rs58073039 | 4 | 88287363 | G | A | -0.014 | 0.002 | 2.16×10^-11^ | 441,291 | 0.298 | 4.04×10^-5^ | 19 | *HSD17B11* |
| SLE | rs58542926 | 19 | 19379549 | T | C | 0.032 | 0.004 | 8.57×10^-19^ | 441,291 | 0.076 | 2.37×10^-5^ | 11 | *TM6SF2* |
| SLE | rs61816766 | 1 | 152319572 | C | T | 0.084 | 0.006 | 1.09×10^-50^ | 441,291 | 0.033 | 3.04×10^-5^ | 14 | *FLG-AS1* |
| SLE | rs61887417 | 11 | 70931788 | A | G | -0.034 | 0.006 | 1.42×10^-9^ | 441,291 | 0.034 | 5.12×10^-5^ | 12 | *SHANK2* |
| SLE | rs6698680 | 1 | 2329661 | G | A | -0.012 | 0.002 | 8.99×10^-10^ | 441,291 | 0.464 | 4.02×10^-5^ | 19 | *RER1* |
| SLE | rs6724965 | 2 | 101440151 | G | A | -0.017 | 0.003 | 1.29×10^-10^ | 441,291 | 0.172 | 2.53×10^-5^ | 12 | *NPAS2* |
| SLE | rs6773343 | 3 | 141825598 | T | C | 0.013 | 0.002 | 5.20×10^-9^ | 441,291 | 0.72 | 2.96×10^-5^ | 14 | *TFDP2* |
| SLE | rs71601787 | 4 | 72866015 | A | G | 0.042 | 0.002 | 3.34×10^-92^ | 441,291 | 0.328 | 3.94×10^-5^ | 183 | *NCR* |
| SLE | rs7178572 | 15 | 77747190 | G | A | -0.014 | 0.002 | 1.62×10^-11^ | 441,291 | 0.711 | 4.01×10^-5^ | 19 | *HMG20A* |
| SLE | rs72665698 | 1 | 41775589 | G | T | 0.014 | 0.002 | 1.99×10^-9^ | 441,291 | 0.223 | 2.69×10^-5^ | 12 | *NCR* |
| SLE | rs73015021 | 19 | 11192915 | G | A | 0.023 | 0.003 | 1.15×10^-14^ | 441,291 | 0.121 | 2.73×10^-5^ | 13 | *NCR* |
| SLE | rs7519574* | 1 | 34726552 | A | G | 0.017 | 0.003 | 2.09×10^-11^ | 441,291 | 0.182 | 2.87×10^-5^ | 13 | *NCR* |
| SLE | rs7528419 | 1 | 109817192 | G | A | 0.019 | 0.002 | 2.41×10^-16^ | 441,291 | 0.225 | 5.05×10^-5^ | 23 | *CELSR2* |
| SLE | rs7569755 | 2 | 118648261 | A | G | 0.014 | 0.002 | 8.03×10^-11^ | 441,291 | 0.292 | 3.76×10^-5^ | 17 | *NCR* |
| SLE | rs7657132 | 4 | 73416601 | G | A | -0.015 | 0.002 | 8.36×10^-13^ | 441,291 | 0.318 | 4.78×10^-5^ | 22 | *ADAMTS3* |
| SLE | rs7699711 | 4 | 69947596 | T | G | -0.029 | 0.002 | 6.97×10^-49^ | 441,291 | 0.455 | 2.31E-04 | 107 | *UGT2B7* |
| SLE | rs77924615 | 16 | 20392332 | A | G | -0.016 | 0.002 | 1.46×10^-10^ | 441,291 | 0.198 | 2.81×10^-5^ | 13 | *PDILT* |
| SLE | rs7930750 | 11 | 14452133 | T | C | -0.060 | 0.002 | 1.10×10^-194^ | 441,291 | 0.368 | 8.87E-04 | 413 | *NCR* |
| SLE | rs804280 | 8 | 11612698 | A | C | 0.013 | 0.002 | 4.43×10^-11^ | 441,291 | 0.582 | 4.55×10^-5^ | 21 | *GATA4* |
| SLE | rs867772 | 1 | 220972343 | G | A | -0.014 | 0.002 | 3.64×10^-11^ | 441,291 | 0.682 | 4.09×10^-5^ | 19 | *MTARC1* |
| SLE | rs9423639 | 10 | 5538801 | T | C | -0.012 | 0.002 | 4.01×10^-8^ | 441,291 | 0.275 | 2.59×10^-5^ | 12 | *NCR* |
| SLE | rs942380 | 6 | 121854778 | G | A | 0.011 | 0.002 | 7.84×10^-9^ | 441,291 | 0.595 | 3.46×10^-5^ | 16 | *NCR* |
| SLE | rs9476310 | 6 | 57767576 | T | C | 0.011 | 0.002 | 1.10×10^-8^ | 441,291 | 0.511 | 3.51×10^-5^ | 16 | *NCR* |
| SLE | rs9569235 | 13 | 55794226 | C | A | -0.013 | 0.002 | 6.10×10^-9^ | 441,291 | 0.285 | 2.96×10^-5^ | 14 | *NCR* |
| SLE | rs960596 | 22 | 41393520 | T | C | 0.012 | 0.002 | 2.23×10^-9^ | 441,291 | 0.34 | 3.45×10^-5^ | 16 | *NCR* |
| SLE | rs9668081 | 12 | 38602911 | T | C | 0.012 | 0.002 | 5.38×10^-9^ | 441,291 | 0.471 | 3.65×10^-5^ | 17 | *NCR* |
| T1DM | rs1048328 | 19 | 51527364 | A | G | 0.028 | 0.004 | 4.12×10^-15^ | 441,291 | 0.914 | 2.06×10^-5^ | 10 | *KLK11* |
| T1DM | rs10908469 | 1 | 155468732 | C | A | 0.016 | 0.002 | 2.20×10^-13^ | 441,291 | 0.741 | 4.39×10^-5^ | 21 | *ASH1L* |
| T1DM | rs11542462 | 16 | 82033810 | A | G | -0.022 | 0.003 | 3.39×10^-14^ | 441,291 | 0.87 | 2.76×10^-5^ | 13 | *SDR42E1* |
| T1DM | rs1229984 | 4 | 100239319 | C | T | -0.047 | 0.006 | 4.85×10^-13^ | 441,291 | 0.048 | 1.02×10^-5^ | 15 | *ADH1B* |
| T1DM | rs17651741* | 15 | 38869666 | A | G | -0.013 | 0.002 | 4.90×10^-8^ | 441,291 | 0.822 | 1.85×10^-5^ | 11 | *LOC107984739* |
| T1DM | rs1800588* | 15 | 58723675 | T | C | -0.030 | 0.002 | 2.65×10^-36^ | 441,291 | 0.793 | 1.10×10^-5^ | 52 | *LIPC* |
| T1DM | rs804280* | 8 | 11612698 | A | C | 0.013 | 0.002 | 4.43×10^-11^ | 441,291 | 0.427 | 4.51×10^-5^ | 21 | *GATA4* |

Summary statistics of instrumental variables were extracted from the largest available genome wide association studies (GWASs) for systemic lupus erythematosus^[1]^ , type 1 diabetes^[2]^ and 25 hydroxyvitamin D level^[3]^. UVMR, univariable mendelian randomization; BIMR, bidirectional mendelian randomization; SLE, systemic lupus erythematosus; T1DM, type 1 diabetes; 25-OHD, 25 hydroxyvitamin D; SNP, single nucleotide polymorphism; Chr, chromosome; EAF, allele frequency of effect allele; SE, standard error of beta; R^2^, variance in exposure explained by each variant, calculated as: R^2^=2*Beta^2*EAF*(1-EAF)/(2*Beta^2*EAF*(1-EAF)+ 2*SE^2*SampleSize*EAF*(1-EAF))^[4]^; F-stat, F-statistic, calculated as: F-statistic=(R^2^/(1-R^2^))*(Sample size -number of instrumental variables -1)/number of instrumental variables)^[5]^; NCR, noncoding region.

* Outliers evaluated by the mendelian randomization pleiotropy residual sum and outlier (MR-PRESSO) test.

[1] Bentham J, Morris DL, Graham DSC, et al. Genetic association analyses implicate aberrant regulation of innate and adaptive immunity genes in the pathogenesis of systemic lupus erythematosus. Nat Genet. 2015;47(12):1457-1464.

[2] Onengut-Gumuscu S, Chen WM, Burren O, et al. Fine mapping of type 1 diabetes susceptibility loci and evidence for colocalization of causal variants with lymphoid gene enhancers. Nat Genet. 2015;47(4):381-386.

[3] Manousaki D, Mitchell R, Dudding T, et al. Genome-wide Association Study for Vitamin D Levels Reveals 69 Independent Loci. *Am J Hum Genet*. 2020;106(3):327-337.

[4] Gill D, Efstathiadou A, Cawood K, Tzoulaki I, Dehghan A. Education protects against coronary heart disease and stroke independently of cognitive function: evidence from Mendelian randomization. Int J Epidemiol 2019;48:1468-1477.

[5] Palmer TM, Lawlor DA, Harbord RM, Sheehan NA, Tobias JH, Timpson NJ, Davey Smith G, Sterne JA. Using multiple genetic variants as instrumental variables for modifiable risk factors. Stat Methods Med Res 2012;21: 223-42.

| **S2 Table. F-statistic and R2 for instrumental variables of T1DM, SLE and 25-OHD level** | | | | |
| --- | --- | --- | --- | --- |
| Expousre | Outcome | *n*.SNPs | R^2^sum | F stat |
| T1DM | SLE | 34 | 2.27×10^-2^ | 36.07 |
| T1DM | 25-OHD level | 33 | 2.46×10^-3^ | 35.26 |
| SLE | T1DM | 14 | 7.53×10^-3^ | 28.65 |
| SLE | 25-OHD level | 38 | 4.11×10^-2^ | 26.13 |
| 25-OHD level | SLE | 70 | 9.62×10^-3^ | 64.45 |
| 25-OHD level | T1DM | 7 | 2.76×10^-4^ | 18.59 |
| *instrumental variables without outliers* | | | | |
| T1DM | SLE | 25 | 8.55×10^-3^ | 18.22 |
| T1DM | 25-OHD level | 33 | 2.44×10^-3^ | 35.96 |
| SLE | T1DM | 12 | 5.55×10^-3^ | 24.58 |
| 25-OHD level | SLE | 69 | 9.59×10^-3^ | 65.18 |
| 25-OHD level | T1DM | 4 | 1.02×10^-4^ | 12.04 |

Outliers evaluated by the mendelian randomization pleiotropy residual sum and outlier (MR-PRESSO) test were eliminated.

SNP, single nucleotide polymorphism; SLE, systemic lupus erythematosus; T1DM, type 1 diabetes; 25-OHD, 25 hydroxyvitamin D; UVMR, Univariable mendelian randomization; BIMR, bidirectional mendelian randomization; F-statistic: a statistic used to evaluate the strength of instruments; R^2^ : the proportion of phenotypic variance explained by all genetic instruments; N.SNPs: Number of SNPs for each exposure; R^2^ sum: Sum of R^2^ of instrumental variables for each exposure.

| **S3 Table . Detailed information of instrumental variables in MVMR** | | | | | | | | | | | | |  |  |  |  |
| --- | --- | --- | --- | --- | --- | --- | --- | --- | --- | --- | --- | --- | --- | --- | --- | --- |
| SNPid | SNP Type | Valid SNP | Chr | Position | Effect Allele | Other Allele | Beta-T1DM | Se-T1DM | *P*-T1DM | Beta-25-OHD | Se-25-OHD | *P*-25-OHD | beta-outcome | se-outcome | *P*-outcome | Gene |
| rs1048328 | 25OHD-Only | T | 19 | 51024108 | G | A | -0.045 | 0.035 | 0.199 | 0.028 | 0.004 | 4.12×10^-15^ | 0.020 | 0.059 | 0.737 | *KLK11* |
| rs10771090 | 25OHD-Only | F | 12 | 24435815 | A | G | 0.025 | 0.019 | 0.188 | -0.011 | 0.002 | 4.32×10^-8^ | 0.083 | 0.030 | 0.005 | *SOX5* |
| rs10908469 | 25OHD-Only | T | 1 | 155498941 | A | C | -0.038 | 0.021 | 7.04×10^-2^ | 0.016 | 0.002 | 2.20×10^-13^ | 0.010 | 0.049 | 0.838 | *ASH1L* |
| rs11203203 | T1DM-Only | T | 21 | 42416077 | G | A | 0.15 | 0.019 | 2.91×10^-15^ | 0.000 | 0.002 | 0.937 | 0.010 | 0.020 | 0.617 | *UBASH3A* |
| rs113010081 | T1DM-Only | T | 3 | 46415921 | T | C | -0.164 | 0.03 | 4.59×10^-8^ | 5.28E-04 | 0.003 | 0.864 | -0.051 | 0.051 | 0.314 | *NCR* |
| rs11542462 | 25OHD-Only | T | 16 | 82000205 | G | A | 0.045 | 0.027 | 9.56×10^-2^ | -0.022 | 0.003 | 3.39×10^-14^ | 0.039 | 0.045 | 0.386 | *SDR42E1* |
| rs12150079 | T1DM-Only | T | 17 | 39869164 | G | A | 0.114 | 0.02 | 1.20×10^-8^ | -0.001 | 0.002 | 0.479 | 0.058 | 0.031 | 0.059 | *ZPBP2* |
| rs12317268 | 25OHD-Only | T | 12 | 21199607 | A | G | -0.028 | 0.025 | 0.263 | -0.019 | 0.003 | 9.15×10^-12^ | 0.030 | 0.046 | 0.521 | *SLCO1B1* |
| rs12416116 | T1DM-Only | T | 10 | 88275897 | C | A | -0.165 | 0.021 | 3.93×10^-15^ | -0.002 | 0.002 | 0.312 | -0.062 | 0.032 | 0.052 | *RNLS* |
| rs1456988 | T1DM-Only | F | 14 | 98021670 | G | T | -0.111 | 0.02 | 2.86×10^-8^ | 0.003 | 0.002 | 0.141 | -0.104 | 0.033 | 0.002 | *NCR* |
| rs151233 | T1DM-Only | T | 16 | 28495107 | C | T | 0.171 | 0.026 | 4.80×10^-11^ | 0.005 | 0.003 | 8.32×10^-2^ | 0.039 | 0.048 | 0.411 | *APOBR* |
| rs1574285 | T1DM-Only | T | 9 | 4283137 | G | T | -0.115 | 0.019 | 1.42×10^-9^ | -0.002 | 0.002 | 0.228 | -0.010 | 0.049 | 0.839 | *GLIS3* |
| rs1701704 | T1DM-Only | T | 12 | 56018703 | T | G | 0.223 | 0.019 | 8.25×10^-32^ | -0.003 | 0.002 | 0.135 | 0.104 | 0.033 | 0.002 | *IKZF4* |
| rs1800588 | 25OHD-Only | T | 15 | 58431476 | C | T | -0.039 | 0.023 | 9.00×10^-2^ | -0.030 | 0.002 | 2.65×10^-36^ | 0.086 | 0.033 | 0.008 | *LIPC* |
| rs1893217 | T1DM-Only | T | 18 | 12809341 | A | G | 0.192 | 0.024 | 1.24×10^-15^ | 1.10E-04 | 0.003 | 0.966 | 0.010 | 0.029 | 0.730 | *PTPN2* |
| rs2111485 | T1DM-Only | F | 2 | 162254026 | A | G | 0.165 | 0.019 | 3.81×10^-18^ | 0.002 | 0.002 | 0.432 | 0.139 | 0.030 | 3.69×10^-6^ | *NCR* |
| rs229533 | T1DM-Only | T | 22 | 37191071 | A | C | 0.107 | 0.019 | 1.79×10^-8^ | 0.001 | 0.002 | 0.535 | 0.068 | 0.026 | 0.010 | *C1QTNF6* |
| rs2611215 | T1DM&25-OHD | T | 4 | 165653115 | A | G | -0.168 | 0.025 | 1.82×10^-11^ | -0.005 | 0.003 | 4.80×10^-2^ | -0.077 | 0.035 | 0.026 | *NCR* |
| rs2934744 | T1DM-Only | T | 1 | 62582374 | A | C | 0.02 | 0.02 | 0.317 | 0.022 | 0.002 | 3.96×10^-26^ | 0.010 | 0.042 | 0.812 | *DOCK7* |
| rs3087243 | T1DM-Only | T | 2 | 203874196 | G | A | -0.178 | 0.019 | 7.36×10^-21^ | -0.002 | 0.002 | 0.344 | -0.083 | 0.032 | 0.009 | *CTLA4* |
| rs3184504 | T1DM-Only | F | 12 | 111446804 | T | C | -0.266 | 0.019 | 1.56×10^-44^ | 0.003 | 0.002 | 0.150 | -0.151 | 0.029 | 1.26×10^-7^ | *SH2B3* |
| rs34293138 | 25OHD-Only | T | 3 | 49541584 | T | C | -0.033 | 0.02 | 9.89×10^-2^ | -0.012 | 0.002 | 3.82×10^-9^ | 0.030 | 0.027 | 0.269 | *NCR* |
| rs34593439 | T1DM&25-OHD | T | 15 | 78942615 | G | A | -0.246 | 0.033 | 9.02×10^-14^ | 0.009 | 0.003 | 7.24×10^-3^ | -0.051 | 0.048 | 0.284 | *CTSH* |
| rs3842727 | T1DM&25-OHD | F | 11 | 2163618 | G | T | 0.687 | 0.023 | 4.89×10^-196^ | -0.004 | 0.002 | 3.2×10^-2^ | -0.020 | 0.026 | 0.442 | *TH* |
| rs41295121 | 25OHD-Only | T | 1 | 6087680 | C | T | -0.653 | 0.112 | 5.53E-09 | 0.008 | 0.009 | 3.83E-01 | -0.211 | 0.141 | 0.136 | *RBM17* |
| rs4820830 | T1DM-Only | T | 22 | 30135102 | C | T | -0.135 | 0.019 | 1.20E-12 | 0.001 | 0.002 | 5.01E-01 | -0.020 | 0.028 | 0.480 | *HORMAD2* |
| rs4849135 | T1DM-Only | T | 2 | 110857502 | T | G | -0.115 | 0.021 | 4.35E-08 | -0.004 | 0.002 | 1.02E-01 | -0.010 | 0.030 | 0.741 | *ACOXL* |
| rs56994090 | T1DM&25-OHD | T | 14 | 100840110 | T | C | -0.129 | 0.019 | 1.13E-11 | 0.004 | 0.002 | 3.34E-02 | -0.020 | 0.025 | 0.423 | *MEG3* |
| rs58542926 | 25OHD-Only | T | 19 | 19268740 | C | T | 0.061 | 0.034 | 7.28E-02 | 0.032 | 0.004 | 8.57E-19 | -0.073 | 0.058 | 0.208 | *TM6SF2* |
| rs6043409 | T1DM-Only | T | 20 | 1635560 | A | G | 0.126 | 0.02 | 2.98E-10 | 6.95E-04 | 0.002 | 7.33E-01 | -0.039 | 0.030 | 0.184 | *SIRPG* |
| rs61839660 | T1DM-Only | F | 10 | 6052734 | C | T | -0.472 | 0.036 | 2.84E-39 | -8.42E-04 | 0.003 | 7.97E-01 | 0.140 | 0.047 | 0.003 | *IL2RA* |
| rs62447205 | T1DM-Only | T | 7 | 50398132 | A | G | -0.117 | 0.021 | 2.53E-08 | -0.002 | 0.002 | 4.29E-01 | -0.041 | 0.035 | 0.237 | *IKZF1* |
| rs6679677 | T1DM-Only | F | 1 | 113761186 | C | A | 0.636 | 0.027 | 1.10E-122 | -0.004 | 0.003 | 2.66E-01 | 0.336 | 0.046 | 4.55×10^-13^ | *PHTF1* |
| rs6691977 | T1DM-Only | T | 1 | 200845831 | T | C | 0.126 | 0.023 | 4.30E-08 | 0.002 | 0.002 | 4.92E-01 | 0.020 | 0.044 | 0.651 | *CAMSAP2* |
| rs6827756 | T1DM-Only | F | 4 | 122263256 | T | C | -0.131 | 0.019 | 5.40E-12 | 0.003 | 0.002 | 1.66E-01 | 0.010 | 0.017 | 0.564 | *KIAA1109* |
| rs7239671 | T1DM-Only | F | 18 | 69856024 | A | G | 0.121 | 0.018 | 1.79E-11 | -0.003 | 0.002 | 1.49E-01 | 0.113 | 0.030 | 1.56×10^-4^ | *CD226* |
| rs72727394 | T1DM&25-OHD | T | 15 | 38554821 | C | T | 0.138 | 0.022 | 3.55E-10 | -0.013 | 0.002 | 1.41E-07 | 0.058 | 0.035 | 0.095 | *RASGRP1* |
| rs72928038 | T1DM-Only | T | 6 | 90267049 | G | A | 0.18 | 0.024 | 6.38E-14 | -0.003 | 0.003 | 2.18E-01 | 0.068 | 0.040 | 0.093 | *BACH2* |
| rs73015021 | 25OHD-Only | T | 19 | 11082239 | A | G | -0.053 | 0.029 | 6.76E-02 | 0.023 | 0.003 | 1.15E-14 | 0.020 | 0.039 | 0.612 | *NCR* |
| rs7528419 | T1DM&25-OHD | T | 1 | 109274570 | A | G | -0.053 | 0.022 | 1.60E-02 | 0.019 | 0.002 | 2.41E-16 | -0.010 | 0.030 | 0.734 | *CELSR2* |
| rs804280 | T1DM&25-OHD | F | 8 | 11755189 | C | A | -0.064 | 0.019 | 7.56E-04 | 0.013 | 0.002 | 4.43E-11 | 0.105 | 0.030 | 4.03×10^-4^ | *GATA4* |
| rs8056814 | T1DM-Only | T | 16 | 75218429 | G | A | 0.278 | 0.031 | 3.03E-19 | -0.005 | 0.004 | 1.89E-01 | 0.030 | 0.050 | 0.556 | *CTRB1* |
| rs9585056 | T1DM-Only | T | 13 | 99429512 | C | T | -0.116 | 0.021 | 3.32E-08 | 0.002 | 0.002 | 4.92E-01 | -0.020 | 0.033 | 0.554 | *LOC107984558* |
| rs9668081 | 25OHD-Only | T | 12 | 38209109 | C | T | 0.037 | 0.019 | 5.15E-02 | 0.012 | 0.002 | 5.38E-09 | -0.020 | 0.037 | 0.582 | *NCR* |

Summary statistics of instrumental variables were extracted from the largest available genome wide association studies (GWASs) for systemic lupus erythematosus^[1]^ , type 1 diabetes^[2]^ and 25 hydroxyvitamin D level^[3]^. MVMR, multivariable mendelian randomization; SLE, systemic lupus erythematosus; T1DM, type 1 diabetes; 25-OHD, 25 hydroxyvitamin D; SNP, single nucleotide polymorphism; Chr, chromosome; SE, standard error of beta; NCR, noncoding region; SNP Type, genetic variants which had both strong correlations with T1DM and 25-OHD level(*P* <0.05) in MVMR were defined as “T1DM&25-OHD”, otherwise defined as “25VHD-Only” or “T1DM-Only”; Valid SNP, the multivariable MR-Lasso method, a pleiotropy robust method to avoid the violations of instrumental variables assumptions, were performed to applies lasso-type penalization to the direct effects of genetic variants on the outcome and identified genetic variants as valid SNPs^[4]^.

[1] Bentham J, Morris DL, Graham DSC, et al. Genetic association analyses implicate aberrant regulation of innate and adaptive immunity genes in the pathogenesis of systemic lupus erythematosus. Nat Genet. 2015;47(12):1457-1464.

[2] Onengut-Gumuscu S, Chen WM, Burren O, et al. Fine mapping of type 1 diabetes susceptibility loci and evidence for colocalization of causal variants with lymphoid gene enhancers. Nat Genet. 2015;47(4):381-386.

[3] Hemani G, Zheng J, Elsworth B, et al. The MR-Base platform supports systematic causal inference across the human phenome. Elife. 2018;7:e34408. Published 2018 May 30. doi:10.7554/eLife.34408.

[4] Grant AJ, Burgess S. Pleiotropy robust methods for multivariable Mendelian randomization. Stat Med. 2021;40(26):5813-583

| **S4 Table. Casual relationships of BIMR among T1DM, SLE, 25-OHD level by approach of MR-IVW, MR-weighted median, MR-weighted mode and MR-Egger** | | | | | | | | | | | |
| --- | --- | --- | --- | --- | --- | --- | --- | --- | --- | --- | --- |
| Exposure | Outcome | *n*.SNPs | MR-IVW | | MR-weighted median | | MR-weighted mode | | MR-Egger | | |
|  |  |  | *P* | OR(95%CI) | *P* | OR(95%CI) | *P* | OR(95%CI) | *P* | *Pinter* | OR(95%CI) |
| *BIMR of SLE AND T1DM* | | | | | | | | | | | |
| T1DM | SLE | 34 | 4.05×10^-3^ | 1.192(1.058-1.344) | 0.372 | 1.038(0.960-1.121) | 0.773 | 0.989(0.921-1.063) | 0.202 | 0.012 | 1.021(0.836-1.247) |
| SLE | T1DM | 14 | 6.16×10^-2^ | 1.053(0.997-1.112) | 3.19×10^-2^ | 1.066(1.006-1.131) | 0.143 | 1.062(0.981-1.150) | 0.321 | 0.331 | 1.037(0.967-1.112) |
| *BIMR of SLE AND 25-OHD level* | | | | | | | | | | | |
| 25-OHD level | SLE | 70 | 2.85×10^-3^ | 0.602(0.431-0.840) | 3.94×10^-3^ | 0.548(0.357-0.842) | 3.61×10^-5^ | 0.430(0.296-0.625) | 6.24×10^-3^ | 0.921 | 0.508(0.317-0.813) |
| SLE | 25-OHD level | 38 | 8.76×10^-2^ | 1.003(0.995-1.007) | 1.36×10^-2^ | 1.004(1.001-1.009) | 8.45×10^-2^ | 1.008(0.992-1.017) | 0.835 | 0.605 | 1.001(0.996-1.039) |
| *BIMR of T1DM AND 25-OHD level* | | | | | | | | | | | |
| T1DM | 25-OHD level | 33 | 3.22×10^-2^ | 0.994(0.989-0.999) | 1.53×10^-2^ | 0.993(0.989-0.998) | 1.52×10^-2^ | 0.994(0.989-0.999) | 0.243 | 0.621 | 0.994(0.985-1.004) |
| 25-OHD level | T1DM | 7 | 0.293 | 0.275(0.024-3.049) | 0.526 | 0.665(0.189-2.343) | 0.879 | 0.228(0.023-2.201) | 4.50×10^-3^ | 0.034 | 19.985(1.063-375.824) |

BIMR, bidirectional mendelian randomization; SLE, systemic lupus erythematosus; T1DM, type 1 diabetes; 25-OHD, 25 hydroxyvitamin D; MR-IVW: mendelian randomization using inverse variance weighted approach; MR-weighted median: mendelian randomization using weighted median method; MR-weighted mode: mendelian randomization using weighted mode; MR-Egger: mendelian randomization using Egger regression; OR, odds ratio; CI, confidence interval; Pinter, the p-value for the MR-Egger intercept test (a low p-value suggests either directional pleiotropy or failure of the InSIDE assumption(Instrument Strength Independent of Direct Effect), and indicates that the IVW estimate is biased)^[1]^.

[1] Bowden J, Davey Smith G, Burgess S. Mendelian randomization with invalid instruments: effect estimation and bias detection through Egger regression. Int J Epidemiol. 2015;44(2):512-525.

| **S5 Table. Sensitivity analysis of directional and horizontal pleiotropy for BIMR and MVMR** | | | | | |
| --- | --- | --- | --- | --- | --- |
| Exposure | Outcome | *n*.SNPs | *P* for MR-PRESSO global test* | *P* for MR-PRESSO distortion test** | *P* for MR Egger intercept test^#^ |
| *BIMR of SLE AND T1DM* | | | | | |
| T1DM | SLE | 34 | <0.001 | 0.022 | 0.012 |
| SLE | T1DM | 14 | 0.008 | 0.353 | 0.331 |
| *BIMR of SLE AND T1DM without outliers* | | | | | |
| T1DM | SLE | 26 | 0.127 | NA | 0.895 |
| SLE | T1DM | 12 | 0.480 | NA | 0.344 |
| *BIMR of SLE AND 25-OHD level* | | | | | |
| 25-OHD level | SLE | 70 | <0.001 | 0.896 | 0.921 |
| SLE | 25-OHD level | 38 | 0.328 | NA | 0.605 |
| *BIMR of SLE AND 25-OHD level without outliers* | | | | | |
| 25-OHD level | SLE | 69 | 0.359 | NA | 0.985 |
| *BIMR of T1DM AND 25-OHD level* | | | | | |
| T1DM | 25-OHD level | 33 | <0.001 | 0.521 | 0.621 |
| 25-OHD level | T1DM | 7 | <0.001 | 1 | 0.034 |
| *BIMR of T1DM AND 25-OHD level without outliers* | | | | | |
| T1DM | 25-OHD level | 32 | 0.603 | NA | 0.627 |
| 25-OHD level | T1DM | 4 | 0.297 | NA | 0.278 |
| *MVMR* | | | | | |
| T1DM&25-OHD level | SLE | 44 | <0.001 | NA | 0.914 |
| *MVMR-Lasso* | | | | | |
| T1DM&25-OHD level | SLE | 34 | 0.446 | NA | 0.605 |

Pleiotropy refers to a genetic variant being associated with multiple risk factors on different causal pathways. If a genetic variant used as an IV is additionally associated with another risk factor for the outcome, then either the second or the third IV assumption is violated, and the variant is not a valid IV. If pleiotropy leads to the genetic variant being associated with the outcome via a confounding variable, then the second assumption would be violated. If pleiotropy leads to an alternative causal pathway from the variant to the outcome not via the exposure of interest, then the third assumption would be violated^[1]^. BIMR, bidirectional mendelian randomization; MVMR, multivariable mendelian randomization; N.SNP is the number of genetic variants. SLE, systemic lupus erythematosus; T1DM, type 1 diabetes; 25-OHD, 25 hydroxyvitamin D.

*MR-PRESSO global test: MR-PRESSO (Mendelian Randomization Pleiotropy Residual Sum and Outlier) is a unified framework that allows for the evaluation of pleiotropy in a standard MR model. The method extends on previous approaches that utilize the general model of multi-instrument MR on summary statistics^[2]^. MR-PRESSO global test could detect pleiotropy of instrumental variables. (a low p-value suggests pleiotropy and the IV assumption is violated)^[3]^.

**MR-PRESSO distortion test: MR-PRESSO distortion test could provide the result of testing of significant distortion in the causal estimate before and after MR-PRESSO correction via outlier removal. NA means”No significant outliers” and need not to test.

#the p-value for the MR-Egger intercept test (a low p-value suggests either directional pleiotropy or failure of the InSIDE(Instrument Strength Independent of Direct Effect) assumption, and indicates that the IVW estimate is biased)^[3]^.

[1] Bowden J, Davey Smith G, Haycock PC, Burgess S. Consistent Estimation in Mendelian Randomization with Some Invalid Instruments Using a Weighted Median Estimator. Genet Epidemiol 2016;40: 304-14.

[2] Verbanck M, Chen CY, Neale B, Do R. Detection of widespread horizontal pleiotropy in causal relationships inferred from Mendelian randomization between complex traits and diseases [published correction appears in Nat Genet. 2018 Aug;50(8):1196]. Nat Genet. 2018;50(5):693-698.

[3] Bowden J, Davey Smith G, Burgess S. Mendelian randomization with invalid instruments: effect estimation and bias detection through Egger regression. Int J Epidemiol. 2015;44(2):512-525.

| **S6 Table. Cochran's Q test for heterogeneity among BIMR analyses** | | | | | | | | | | |
| --- | --- | --- | --- | --- | --- | --- | --- | --- | --- | --- |
| Exposure | Outcome | *n*.SNPs | MR-IVW | | |  | MR-Egger | | | |
|  |  |  | Q.stat | Q_df | Q.stat_*P* |  | Q.stat | Q_df | Q.stat_*P* | I_GX_^2^* |
| *BIMR of SLE AND T1DM* | | | | | | | | | | |
| T1DM | SLE | 34 | 31.949 | 33 | 0.519 |  | 25.651 | 32 | 0.779 | 0.990 |
| SLE | T1DM | 14 | 27.344 | 13 | 0.011 |  | 27.147 | 12 | 0.007 | 0.989 |
| *BIMR of SLE AND T1DM without outliers* | | | | | | | | | | |
| T1DM | SLE | 25 | 32.481 | 24 | 0.116 |  | 32.477 | 23 | 0.091 | 0.981 |
| SLE | T1DM | 12 | 11.964 | 11 | 0.366 |  | 10.982 | 10 | 0.359 | 0.988 |
| *BIMR of SLE AND 25-OHD level* | | | | | | | | | | |
| 25-OHD level | SLE | 70 | 116.949 | 69 | 2.79×10^-4^ |  | 115.244 | 68 | 3.06×10^-4^ | 0.995 |
| SLE | 25-OHD level | 38 | 68.998 | 68 | 0.443 |  | 68.998 | 67 | 0.410 | 0.995 |
| *BIMR of SLE AND 25-OHD level without outliers#* | | | | | | | | | | |
| 25-OHD level | SLE | 69 | 37.489 | 37 | 0.447 |  | 37.213 | 36 | 0.413 | 0.989 |
| *BIMR of T1DM AND 25-OHD level* | | | | | | | | | | |
| T1DM | 25-OHD level | 33 | 75.891 | 32 | 1.99×10^-5^ |  | 75.890 | 31 | 1.24×10^-5^ | 0.989 |
| 25-OHD level | T1DM | 7 | 46.313 | 6 | 2.56×10^-8^ |  | 12.446 | 5 | 2.92×10^-2^ | 0.986 |
| *BIMR of T1DM AND 25-OHD level without outliers* | | | | | | | | | | |
| T1DM | 25-OHD level | 32 | 51.308 | 31 | 0.012 |  | 50.840 | 30 | 0.010 | 0.990 |
| 25-OHD level | T1DM | 4 | 4.364 | 3 | 0.225 |  | 0.881 | 2 | 0.644 | 0.985 |

The Q statistic has a chi-squared distribution on N-1 degrees of freedom(df) under the null hypothesis that all genetic variants are valid IVs and the same causal effect is identified by all variants.^[1]^ N.SNP is the number of genetic variants. BIMR, bidirectional mendelian randomization; SLE, systemic lupus erythematosus; T1DM, type 1 diabetes; 25-OHD, 25 hydroxyvitamin D; MR-IVW: mendelian randomization using inverse variance weighted approach; MR-Egger: mendelian randomization using Egger regression.

* An adapted I^2^ statistics to quantify the strength of NOME (No Measurement Error) violation for instruments used for MR-Egger regression.

# In MR analysis with SLE as exposure and 25-OHD level as result, there were no significant outliers identified by the MR-PRESSO test.

[1] Bowden J, Davey Smith G, Haycock PC, Burgess S. Consistent Estimation in Mendelian Randomization with Some Invalid Instruments Using a Weighted Median Estimator. Genet Epidemiol 2016;40: 304-14.

| **S7 Table. Casual relationships of BIMR among T1DM, SLE, 25-OHD level without outliers** | | | | | | | | | | | |
| --- | --- | --- | --- | --- | --- | --- | --- | --- | --- | --- | --- |
| Exposure | Outcome | *n*.SNPs | MR-IVW | | MR-weighted median | | MR-weighted mode | | MR-Egger | | |
|  |  |  | *P* | OR(95%CI) | *P* | OR(95%CI) | *P* | OR(95%CI) | *P* | *Pinter* | OR(95%CI) |
| *BIMR of SLE AND T1DM* | | | | | | | | | | | |
| T1DM | SLE | 25 | 1.17×10^-6^ | 1.264(1.150-1.389) | 9.68×10^-3^ | 1.181(1.041-1.339) | 0.371 | 1.118(0.880-1.419) | 0.254 | 0.895 | 1.278(0.847-1.928) |
| SLE | T1DM | 12 | 6.88×10^-2^ | 1.042(0.996-1.089) | 0.223 | 1.038(0.978-1.103) | 0.272 | 1.049(0.967-1.138) | 0.336 | 0.344 | 1.037(0.964-1.115) |
| *BIMR of SLE AND 25-OHD level** | | | | | | | | | | | |
| 25-OHD level | SLE | 69 | 2.62×10^-3^ | 0.597(0.427-0.835) | 3.67×10^-3^ | 0.548(0.357-0.842) | 1.52×10^-5^ | 0.431(0.303-0.614) | 6.88×10^-3^ | 0.985 | 0.509(0.317-0.818) |
| *BIMR of T1DM AND 25OHD level* | | | | | | | | | | | |
| T1DM | 25-OHD level | 32 | 3.05×10^-2^ | 0.995(0.991-0.999) | 2.32×10^-2^ | 0.994(0.988-0.999) | 1.43×10^-2^ | 0.994(0.989-0.998) | 0.108 | 0.627 | 0.993(0.986-1.001) |
| 25-OHD level | T1DM | 4 | 0.106 | 0.301(0.070-1.288) | 2.08×10^-2^ | 0.173(0.039-0.766) | 0.188 | 0.130(0.012-1.372) | 0.635 | 0.278 | 0.553(0.048-6.348) |

BIMR, bidirectional mendelian randomization; SLE, systemic lupus erythematosus; T1DM, type 1 diabetes; 25-OHD, 25 hydroxyvitamin D; MR-IVW: mendelian randomization using inverse variance weighted approach; MR-weighted median: mendelian randomization using weighted median method; MR-weighted mode: mendelian randomization using weighted mode; MR-Egger: mendelian randomization using Egger regression; OR, odds ratio; CI, confidence interval; Pinter, the p-value for the MR-Egger intercept test (a low p-value suggests either directional pleiotropy or failure of the InSIDE(Instrument Strength Independent of Direct Effect) assumption, and indicates that the IVW estimate is biased)^[1]^.

*In MR analysis with SLE as exposure and 25-OHD level as result, there were no significant outliers identified by the MR-PRESSO test.

[1] Bowden J, Davey Smith G, Burgess S. Mendelian randomization with invalid instruments: effect estimation and bias detection through Egger regression. Int J Epidemiol. 2015;44(2):512-525.
